# Mechanosensitive biochemical imprinting of the talin interaction with DLC1 regulates RhoA activity and cardiomyocyte remodeling

**DOI:** 10.1126/sciadv.adt6083

**Published:** 2025-09-05

**Authors:** Emilie Marhuenda, Ioannis Xanthis, Poppy O. Smith, Aishwarya Prakash, Till Kallem, Pragati Pandey, Darren Graham Samuel Wilson, Amar Azad, Megan Richter, Davor Pavlovic, Katja Gehmlich, Giuseppe Faggian, Pamela Swiatlowska, Elisabeth Ehler, James Levitt, Simon P. Poland, Simon Ameer-Beg, Benjamin T Goult, Thomas Iskratsch

**Affiliations:** ^1^ School of Engineering and Materials Science, Queen Mary University of London, UK.; ^2^ Université de Bordeaux, CNRS, Bordeaux INP, CBMN, UMR 5248, F-33600 Pessac, France.; ^3^ Department of Biochemistry, Cell and Systems Biology, Institute of Systems, Molecular and Integrative Biology, University of Liverpool, Crown Street, Liverpool L69 7ZB, UK.; ^4^ Institute of Cardiovascular Sciences, University of Birmingham, Birmingham B15 2TT, UK.; ^5^ Division of Cardiovascular Medicine, Radcliffe Department of Medicine and British Heart Foundation Centre of Research Excellence Oxford, University of Oxford, Oxford OX3 9DU, UK.; ^6^ Divisione Ospedaliero Universitaria Cardiochirurgia Verona, Verona, Italy.; ^7^ National Heart and Lung Institute, Imperial College London, UK.; ^8^ School of Cardiovascular and Metabolic Medicine and Sciences, King’s College London, UK.; ^9^ School of Cancer and Pharmaceutical Sciences, King’s College London, UK.

## Abstract

During heart disease, the cardiac extracellular matrix (ECM) undergoes a structural and mechanical transformation. Cardiomyocytes sense the mechanical properties of their environment, leading to phenotypic remodeling. A critical component of the ECM mechanosensing machinery, including the protein talin, is organized at the cardiomyocyte costamere. Our previous work indicated a different talin tension, depending on the ECM stiffness, but the effects on downstream signaling remained elusive. Here, we identify that the talin interacting proteins DLC1 (deleted in liver cancer 1), RIAM (Rap1-interacting adaptor molecule), and paxillin each preferentially bind to talin at a specific ECM stiffness, this interaction is preserved in the absence of tension, and the interaction is regulated through focal adhesion kinase signaling. Moreover, DLC1 regulates cardiomyocyte RhoA activity in a stiffness-dependent way, whereby the loss of DLC1 results in myofibrillar disarray. Together, this study demonstrates a mechanism of imprinting mechanical information into the talin interactome to fine-tune RhoA activity, with impacts on cardiac health and disease.

## Teaser

Cardiomyocytes’ sensing of extracellular matrix stiffness is imprinted in the talin interactome and regulates their phenotype.

## INTRODUCTION

Cardiomyocytes are the contractile cells in the heart, and their proper function is regulated through a complex signaling network that includes electrical, chemical, and mechanical signals (*1*). The mechanical signals are varied and include pressure and stretch from the filling of the heart with blood and the stiffness of the extracellular matrix (ECM). The ECM stiffness changes during aging and in heart disease, where changing ECM, protein expression, and excessive cross-linking of collagen through lysyl oxidases (LOX) and LOX-like enzymes can lead to a stiffening of the heart, cardiomyocyte phenotypic changes, and heart failure with preserved ejection fraction (*2*–*8*). Cardiomyocytes attach to the ECM through so-called costameres, rib-like structures at the level of the sarcomeric Z-disc, which contain integrins as well as other multimolecular complexes for adhesion (e.g., the dystrophin glycoprotein complex) and/or signaling (*9*). The cardiomyocyte integrin adhesion contains many of the proteins that are also found in focal adhesions, including talin and vinculin. These proteins connect to the actin cytoskeleton and through actin cross-linkers, such as α-actinin and plectin, further to the sarcomeric Z-disc (*10*).

Previous studies, including our work, documented the role of ECM stiffness in determining the contraction dynamic and contractile work of cardiomyocytes (*11*–*16*). Moreover, our previous work suggested that cardiomyocytes can sense the ECM stiffness at integrin adhesions, through a combination of myofibrillar and nonmyofibrillar contractions (*16*). Nonmuscle myosin pulling on actin generates a baseline tension on integrin adhesions during diastole, to which the forces from the myofibrillar contractions are cyclically added. Depending on the stiffness of the ECM and the level of nonmyofibrillar contraction, this leads to a different amount of tension on the integrin adhesion protein talin. Overall, the resulting forces are insufficient to reach the threshold to extend the helix bundles in the rod domain of talin on embryonic heart stiffness but lead to cyclic extensional strain on healthy adult heart stiffness and constant extensional strain on fibrotic stiffness. Moreover, nonmuscle myosin is increasingly active at the costameres in heart disease, suggesting that the baseline tension is further modulated in heart disease with influences on cardiomyocyte function and disease progression.

Talin is increasingly regarded as a key mechanical sensor molecule (*17*–*19*). It contains a head domain that binds to the integrins, as well as a rod domain, which is composed of 13 helical bundles (R1 to R13) that include actin binding sites and further bind to a substantial number of other proteins (*17*). All rod domains can unfold under physiological forces (between 5 and 12 pN) and refold once tension is released (*19*). This opening and closing can lead to not only a remarkable extension of the protein to over 800 nm (*19*), but also to the exposure of cryptic binding sites, most prominently for vinculin (*20*), which can then lead to adhesion reinforcement. Our previous work showed enriched localization of vinculin to force transduction sites (in this case, nanopillars that were being pulled by the cardiomyocytes), and this enrichment in localization increased with pillar stiffness, suggesting adhesion reinforcement (*16*).

However, apart from vinculin, other force-dependent interactions have been reported for talin, including Rap1-interacting adaptor molecule (RIAM; also known as amyloid-β precursor protein binding family B), which binds to the talin R3 domain only in its folded form and, thus, in a mutually exclusive way to vinculin, as well as to the R8 domain (*21*). Similarly, the protein “deleted in liver cancer 1” (DLC1) binds to the R8 domain in the folded state but dissociates once it is unfolded under force (*22*). DLC1 is an attractive candidate for downstream mechanosignaling in cardiomyocytes. DLC1 is located at the plasma membrane and adhesions, where it acts as a Rho guanosine triphosphatase (GTPase) activating protein (RhoGAP; i.e., negative regulator of RhoA activity) and hence could regulate RhoA activity in relation to changes to the mechanical environment (*22*–*24*). RhoA activity has been linked to cardiomyocyte hypertrophy and increased fibrotic response to chronic pressure overload (*25*–*27*). On the other hand, RhoA is also essential for early heart development (*28*) and has protective effects after cardiac injury, whereby RhoA promotes cardiomyocyte survival (*29*) and delays the transition to heart failure (*25*). Together, this indicates the need for a tight spatial and temporal regulation of RhoA activity in response to diverse chemical (e.g., downstream of hypertrophic stimuli acting on G protein–coupled receptors, such as phenylephrine, endothelin-1, or angiotensin II) (*30*) and mechanical signals.

Here, we investigate the cardiomyocyte mechanosignaling pathway downstream of talin. We find that stiffness regulates differential binding of the three talin R8 domain binding partners: DLC1, RIAM, and paxillin. We identify that the regulation of the interactions is regulated through direct competition for binding, as well as focal adhesion kinase (FAK) activity. Moreover, the interaction remains imprinted even after tension is released, and the changes to the interactions are also evident in vivo. Last, we identify that DLC1 is a major RhoGAP at cardiomyocyte adhesions, and, consequentially, a knockdown of DLC1 results in stiffness-dependent cytoskeletal disruptions and alteration of cardiomyocyte mechanics. In summary, our data suggest a mechanism of mechanotransduction and mechanosensitive biochemical imprinting that depends on talin stretching and altered talin interactions. Overall, we find that mechanosensitive biochemical imprinting modifies talin interactions through integrin-related kinase signaling and competition for binding to the talin R8 domain. Together, this determines cardiomyocyte function through regulating the levels of active RhoA via DLC1, with implications for pathological cardiac remodeling.

## RESULTS

### DLC1 is the major RhoGAP in cardiomyocyte adhesions and binds talin in a stiffness-dependent way

**Fig. 1.**
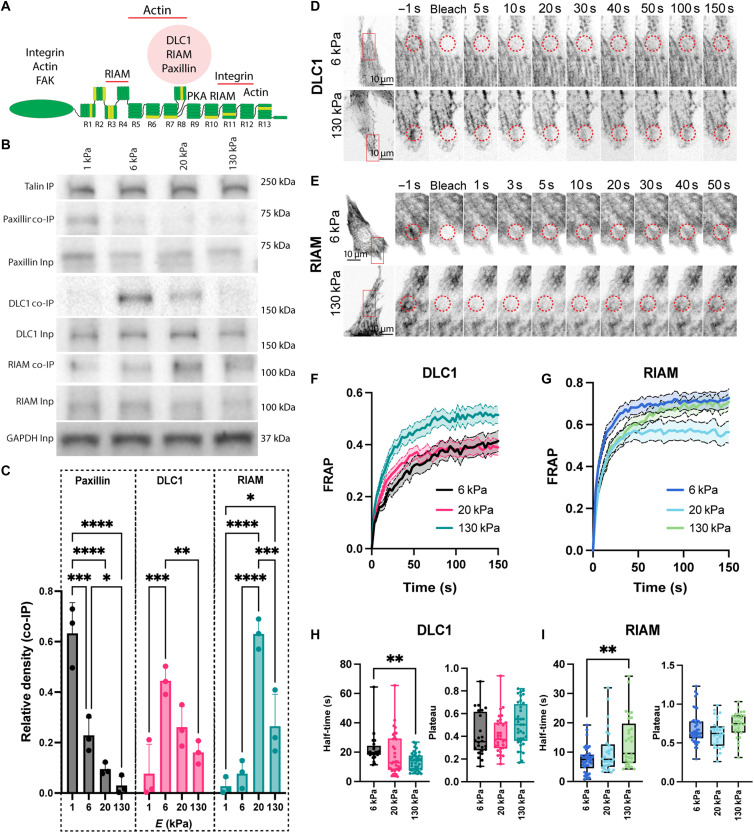
DLC1 and RIAM bind to talin in a stiffness-dependent way. (**A**) Schematic of talin 1 with protein interactions [only interactions that are relevant for this manuscript are shown, see also Calderwood *et al.* (*72*)]. Vinculin binding helices are shown in light green. (**B**) Coimmunoprecipitation (co-IP) analysis for talin interactions. Neonatal rat cardiomyocytes were cultured on polydimethylsiloxane (PDMS) surfaces with the indicated stiffness for 7 days before immunoprecipitation with an anti-talin antibody. Samples were then subjected to Western blotting and probed with antibodies against paxillin, RIAM and DLC1, indicating preferential binding at 1 kPa (paxillin), 6 kPa (DLC1), and 20 kPa (RIAM). Inp, input; IP, immunoprecipitation. (**C**) Quantification of the data in (B) from three independent biological repeats. (**D** and **E**) Fluorescence recovery after photobleaching (FRAP) assays of DLC1 (D) and RIAM (E) in neonatal rat cardiomyocytes, from three independent biological repeats with 25 (DLC1 6 kPa), 36 (DLC1 20 kPa), 36 (DLC1 130 kPa), 33 (RIAM 6 kPa), 29 (RIAM 20 kPa), and 31 (RIAM 130 kPa) cells quantified per condition. (**F** and **G**) Average recovery curves (means + SEM). (**H**) Quantification recovery half-time and plateau (mobile fraction) for DLC1 and (**I**) RIAM. *P < 0.05; **P < 0.01; ***P < 0.001; ****P < 0.0001, one-way analysis of variance (ANOVA) with Tukey correction for multiple comparisons (C) or unpaired *t* test (H and I).

We previously found that cardiomyocyte sensing of ECM stiffness resulted in different modes of talin stretching (no stretching versus cyclic versus static) (*16*). All talin rod domains can unfold and refold under physiological forces, and several binding partners have been shown to bind in a force-dependent way, including RIAM, DLC1, and adenosine 3′,5′-monophosphate–dependent protein kinase (also known as Protein Kinase A, or PKA; Fig. 1A) (*19*–*21*, *31*). Because of its mechanosensitive binding to talin and its role in regulating RhoA (*22*), we hypothesized that DLC1 could be involved in downstream signaling. To test whether DLC1 binding to talin could regulate the mechanical signaling in the heart, we first confirmed its expression in cardiomyocytes. Single-cell and single-nucleus RNA sequencing (RNA-seq) datasets from fetal and adult human hearts (*32*, *33*), as well as from postnatal mouse hearts (*34*), all suggested that DLC1 is consistently expressed in cardiomyocytes at the highest level of all adhesion-localized RhoA, Rac, or Cdc42 guanine nucleotide exchange factors (GEFs) and GTPase activating proteins (GAPs) [fig. S1, A to C; whereby adhesion localization of these molecules was previously determined by Muller *et al.* (*35*)]. Similarly, our bulk RNA-seq dataset of control mouse hearts also indicated that DLC1 is the major adhesion-localized small GTPase regulator in the heart (fig. S1D) (*36*). In agreement with this, we found adhesion staining of DLC1 in neonatal rat cardiomyocytes (NRC; fig. S1E). We further detected DLC1 in wild-type (WT) mouse hearts by Western blotting and immunostaining, showing a costameric pattern (fig. S1, F and G). DLC1 is up-regulated in the muscle LIM protein (MLP) knockout mouse, an established model for dilated cardiomyopathy (*37*). However, in this case, no clear costameric pattern was detected (fig. S1, F to I).

Having confirmed cardiomyocyte-specific expression, we next wanted to test whether DLC1 binding to talin might be regulated through ECM stiffness. For this, we first performed coimmunoprecipitation (co-IP) with an anti-talin antibody. Because DLC1 shares an overlapping binding site on the talin R8 domain with RIAM and paxillin (*38*), we decided to simultaneously probe the interactions of paxillin and RIAM. Paxillin is well described as a costameric protein in the heart and interacts, among others, with FAK (*39*, *40*). Similar to DLC1, both RIAM and paxillin were found to be expressed in cardiomyocytes (both ventricular and atrial) when we reanalyzed single-cell RNA-seq data from human hearts (fig. S2) (*32*). Immunostaining further showed that RIAM is expressed at costameres, whereby expression is increased in MLP knockout mice and human heart disease (fig. S3).

We found a strong stiffness dependence in the co-IP of all three proteins, whereby each protein had a specific optimal stiffness for interacting with talin (Fig. 1, B and C). Paxillin interacted strongest at embryonic heart stiffness (1 kPa), DLC1 interacted strongest at healthy adult heart stiffness (6 kPa), and RIAM interacted strongest at elevated stiffness (20 and 130 kPa).

**Fig. 2.**
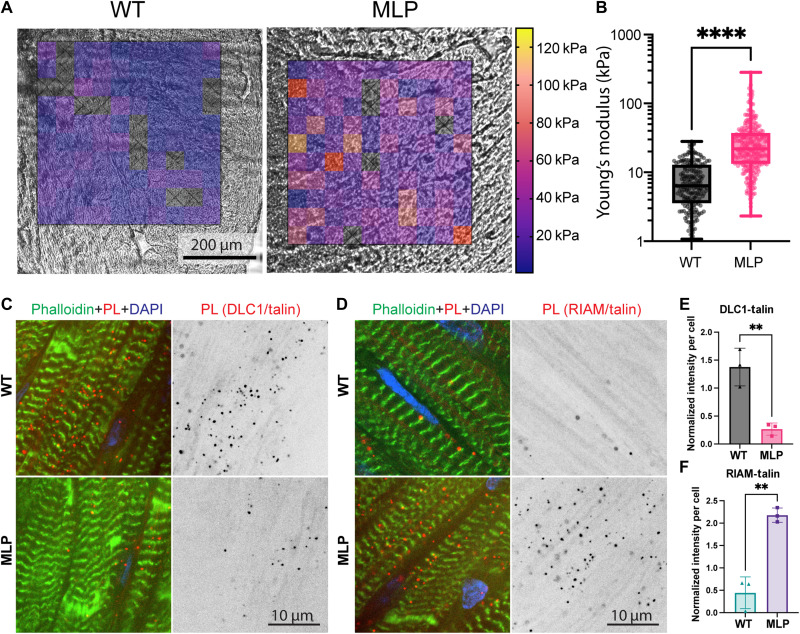
DLC1 and RIAM binding to talin is altered in vivo in heart disease. (**A** and **B**) Nanoindentation measurement of sections from WT and MLP knockout hearts. Sections (20-μm thick) of *n* = 3 hearts each were measured using a nanoindenter (tip radius = 49 μm, stiffness = 0.49 N/m, and indentation depth = 2 μm). (A) Stiffness map overlaid over the bright-field image. Empty squares indicate areas where the measurement failed, possibly due to uneven topography. (B) Quantification. ****P < 0.0001, unpaired *t* test after logarithmic transformation of the (log-normal distributed) data. (**C** to **F**) In situ proximity ligation (PL) assays were performed on sections from WT and MLP knockout hearts with DLC1 and talin (C) and RIAM and talin antibodies (D); *n* = 3 animals each. (E and F) Integrated intensity of proximity labels were normalized by the number of nuclei per image, and >5 images were quantified per heart. Data are presented as mean per animal, normalized by the mean of all images of the respective repeat. **P < 0.01, unpaired *t* test.

To test for the state of talin interactions in cellulo, we performed fluorescence recovery after photobleaching (FRAP) experiments to investigate the stiffness-dependent dynamics of DLC1 and RIAM at adhesion sites (Fig. 1, D to I). While DLC1 has been reported to bind to other adhesion proteins, such as tensin (*41*), and RIAM can also bind to the talin R3 and R11 domains (*21*), we nevertheless observed a slower turnover of DLC1 at healthy adult stiffness (i.e., stronger binding to the adhesion sites at 6 kPa). Conversely, RIAM exhibited a slower turnover at a fibrotic stiffness of 130 kPa (Fig. 2, F to I), in agreement with the immunoprecipitation data. Half-times on 20-kPa polydimethylsiloxane (PDMS) were in between the values for 6 and 130 kPa for both RIAM and DLC1. The plateau was unchanged for DLC1 and RIAM, suggesting no change in the immobile fraction (i.e., the stable binding molecular pool that does not undergo exchange during the time frame of observation; Fig. 1, H and I). On one hand, this result confirmed the differential interaction in cellulo. On the other hand, the fact that talin is in a tension-free state during the immunoprecipitation suggested that the competitive interactions might be further imprinted, i.e., stabilized through other mechanisms, such as posttranslational modifications.

### DLC1 and RIAM binding to talin is differentially regulated in heart disease

To further test the relevance of the findings, we performed in situ proximity ligation assays (PLAs) using combinations of DLC1 and talin, as well as RIAM and talin antibodies (Fig. 2 and fig. S4A for the schematic). We performed the assays in heart sections of MLP knockout mice and WT controls. These tissues had mean Young’s moduli of 8.5 ± 0.4 kPa for WT and 31.6 ± 2.0 kPa for MLP knockout hearts, as measured by nanoindentation. Peak Young’s moduli were measured at >100 kPa in the diseased samples (Fig. 2, A and B). Overall, the nanoindentation measurement aligned well with previous data [e.g., from a recent study from mouse hearts (*42*); further measurements with different methods are reviewed here (*1*)]. These measurements also matched the range of stiffnesses that showed altered binding of DLC1 and RIAM to talin (Fig. 1). The PLA resulted in clusters of labeled colocalizations, which were primarily found close to the cell membranes, often at the level of the I-band, suggesting a costameric localization (Fig. 2, C to F, and fig. S4B for negative and positive controls). DLC1 interactions with talin were down-regulated and RIAM interactions with talin were increased in the MLP knockout hearts, suggesting a potential stiffness-dependent regulation in agreement with the findings from the in vitro experiments. Notably, in contrast to the costameric RIAM staining in WT mice (fig. S3G), we could not detect RIAM interaction with talin in the proximity ligation experiment (Fig. 2, C to D), suggesting that, in the WT hearts, costameric RIAM might be interacting with proteins other than talin.

### DLC1 and RIAM binding is regulated through FAK signaling

**Fig. 3.**
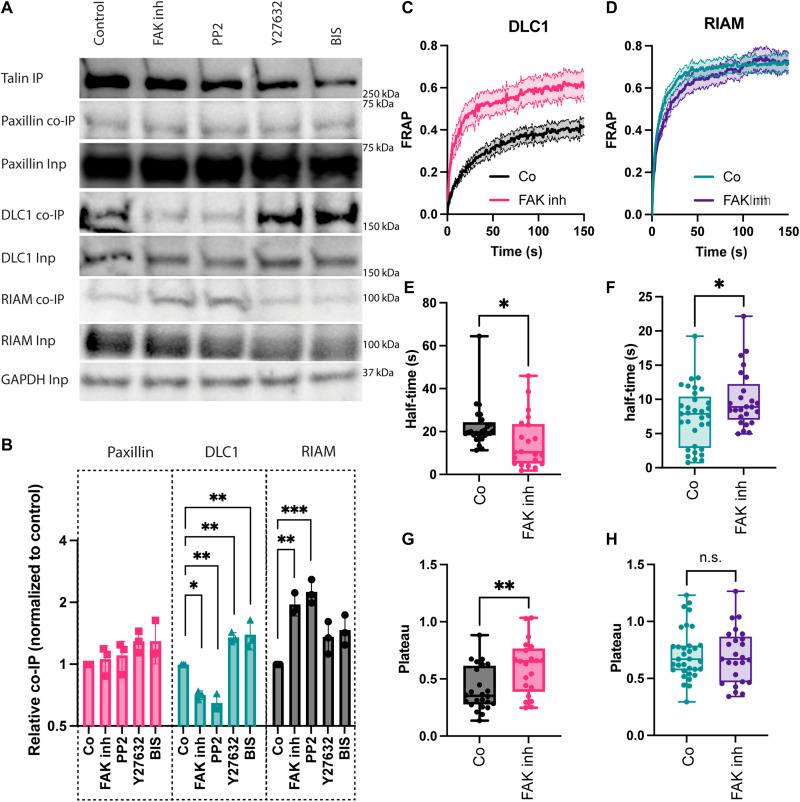
The talin interactome is regulated through phosphorylation. (**A**) Co-IP analysis for talin interactions. Neonatal rat cardiomyocytes were cultured on PDMS with 6 kPa stiffness for 7 days, treated with the indicated inhibitors, and immunoprecipitated with an anti-talin antibody. Samples were then subjected to Western blotting and probed with antibodies against paxillin, RIAM, and DLC1, indicating changes in the talin interactions after FAK and SFK inhibition (inh). (**B**) Quantification from three independent repeats. (**C** to **H**) FRAP assays of DLC1 (C, E, and G) and RIAM (D, F, and H) in neonatal rat cardiomyocytes from three independent biological repeats, with 25 [DLC1 6 kPa control (Co); see Fig. 1], 21 (DLC1 6 kPa FAK inh), 33 (RIAM 6 kPa Co; see Fig. 1), and 25 (RIAM 6 kPa FAK inh) cells quantified per condition. (C and D) Average recovery curves (means + SEM). (E to H) Quantification of recovery half-time and plateau (mobile fraction) for DLC1 (E and G) and RIAM (F and H). *P < 0.05; **P < 0.00; ***P < 0.001, one-way ANOVA with Tukey correction for multiple comparisons (B) or unpaired *t* test (E to H). n.s., not significant.

Because the stiffness dependence of talin interactions in cardiomyocytes persisted in the absence of tension, we hypothesized that the interactions might be regulated through integrin-related phosphorylation pathways, such as Src family kinases (SFK), FAK, Rho kinase (ROCK), or protein kinase C (PKC). To test this, we pretreated the cardiomyocytes on healthy adult heart stiffness (6 kPa) with the respective inhibitors [protein phosphatase 2 (PP2), FAK inhibitor, Y27632, and bisindolylmaleimide I (BIS I)] before cell lysis and performed the co-IP in the presence of the inhibitors (Fig. 3, A and B). Confirming our hypothesis, SFK and FAK inhibition altered the talin interactions, whereby the bound fraction of RIAM was increased and the interaction with DLC1 was simultaneously reduced, suggesting that the competition between DLC1 and RIAM for the talin binding site (TBS) was dependent on integrin-related signaling through SFKs and FAK. Notably, we did not find any changes to paxillin binding, and the inhibition of ROCK and PKC showed only minor effects (both slightly increasing the binding of DLC1 and RIAM, compared to the control). Western blotting for active phosphorylated FAK indicated that FAK activity was highest at 6 kPa (fig. S5, A and B). In contrast, a phospho-SFK (pSFK) antibody did not show significant stiffness-dependent differences. Notably, the pSFK bands did not overlap with Src (fig. S5, C and D). Because of the activity of PP2 against multiple SFKs and the cross-reactivity of the pSFK antibody against other Src family members (Lyn, Fyn, Lck, Yes, and Hck), we decided to focus on FAK for further experiments. Again, we turned to FRAP experiments to observe the changes of DLC1 and RIAM binding to cell adhesions in cellulo, after FAK inhibition (Fig. 3, C to H). The changes to the protein dynamics agreed with the results from the co-IP experiments, showing an increased half-time of recovery for RIAM and a reduced half-time for DLC1 after FAK inhibition (Fig. 3, E and F). For DLC1, the plateau was also increased, indicating a reduced immobile fraction, although it did not show any significant change for RIAM. Because of the disruptive effect of SFK inhibition on the cardiomyocyte adhesion morphology, we were not able to reliably evaluate the changes to half-time or plateau after PP2 treatment.

### DLC1 and RIAM directly compete for the binding to R8 in cardiomyocytes

**Fig. 4.**
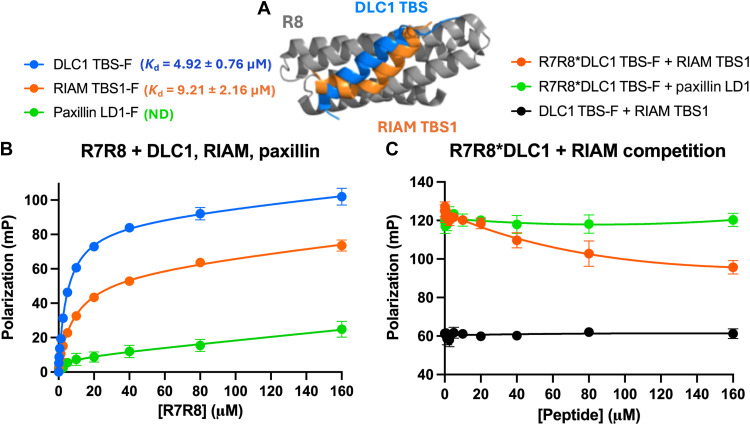
DLC1 and RIAM directly compete for talin binding in vitro. FP binding and competition experiments of purified talin R7R8 with DLC1 TBS, RIAM TBS1, and paxillin LD1 synthetic peptides. (**A**) Overlay of the crystal structures of talin 1 R8 (gray) in complex with DLC1 TBS (blue, pdb 5FZT) and RIAM TBS1 (orange, pdb 4W8P) peptides, showing that the binding site for the two peptides overlap. (**B**) FP binding curves of purified R7R8 with TBS-F (blue, *K*_d_ = 4.92 ± 0.76 μM), RIAM TBS1 (orange, *K*_d_ = 9.21 ± 2.16 μM), or paxillin LD1 [green, not determined (ND)]. For clarity, the fluorescein-labeled peptides are designated with a “-F” in the figure. (**C**) FP competition experiment of R7R8 complexed with DLC1 TBS-F in competition with unlabeled RIAM TBS1. Increasing amounts of unlabeled RIAM peptide outcompetes the labeled DLC1 TBS-F as seen by a decrease in polarization [orange, inhibitory constant (*K*_i_) = 90 ± 48 μM]. The R7R8-DLC1 TBS-F complex could not be outcompeted with unlabeled paxillin LD1 (green). No changes in signal were observed when DLC1 TBS-F was titrated with unlabeled RIAM TBS1 peptide (black).

RIAM can bind the R3, R8, and R11 talin domains, but the FRAP experiments indicated a competition with DLC1 for the binding to the R8 domain, with opposite trends for RIAM and DLC1 binding to talin downstream of increased ECM stiffness and FAK inhibition. DLC1, RIAM, and paxillin bind to the same surface on the R8 domain, indicating that these interactions should be competitive, although this has never been experimentally validated. To directly measure the binding of DLC1, RIAM, and paxillin to talin and a potential competition of their binding, we first opted for in vitro binding experiments. For this, we tested the interaction of synthetic (unphosphorylated) peptides for TBS of DLC1, RIAM (TBS1), and paxillin (LD1) to a purified talin 1 R7R8 domain protein (Fig. 4). The peptides were labeled with fluorescein for fluorescence polarization (FP) experiments to (i) measure binding to R7R8 and (ii) test for competition between DLC1 TBS and RIAM TBS1 peptides. FP binding experiments showed that DLC1 TBS and RIAM TBS1 have dissociation constant (*K*_d_) values of 4.9 and 9.2 μM, respectively, while the *K*_d_ of paxillin LD1 could not be determined over the same range due to low affinity (Fig. 4B and fig. S6).

For the competition experiments, DLC1 TBS-fluorescein (TBS-F) synthetic peptide was complexed with R7R8 and unlabeled RIAM TBS1 peptide was added in increasing amounts. In this experiment, the displacement of the labeled DLC1 peptide, upon addition of an unlabeled peptide, would result in a decrease in measured FP signal, indicating competitive binding (Fig. 4C). As the concentration of unlabeled RIAM TBS1 peptide increased, a decrease in FP signal was observed, as bound DLC1 peptide was displaced, confirming direct competition between these two peptides for binding to R8. In contrast, a similar experiment with an unlabeled paxillin LD1 peptide, at the same concentrations as RIAM TBS1, was unable to compete effectively with DLC1 TBS-F due to its much lower *K*_d_. These biochemical experiments confirm direct competition between DLC1 TBS and RIAM TBS1 for binding to talin 1 R8.

**Fig. 5.**
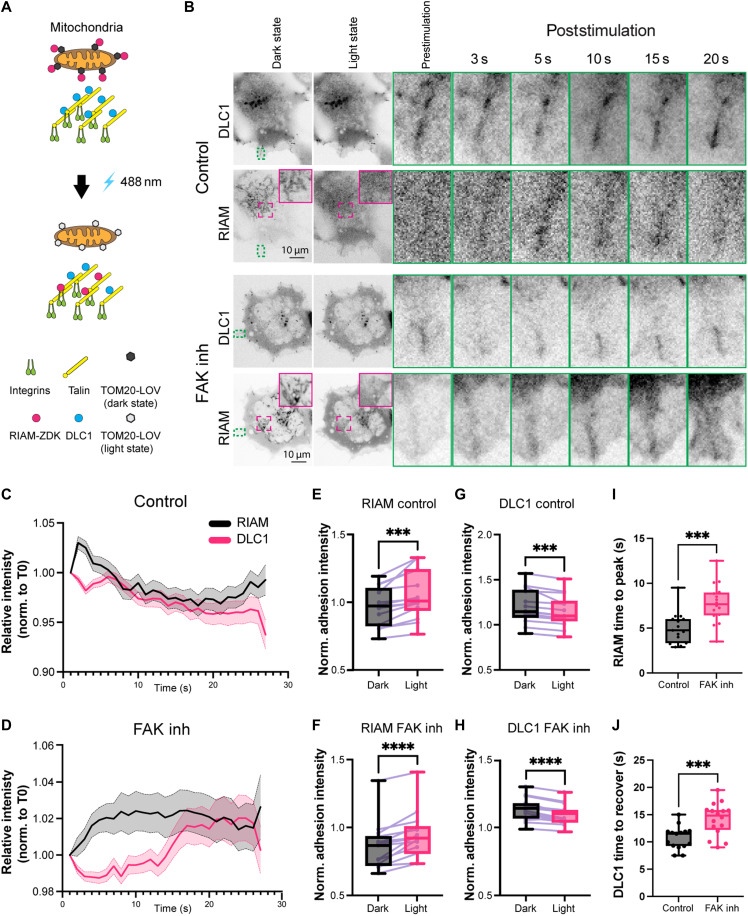
DLC1 and RIAM directly compete for talin binding in cells. (**A**) Schematic of the LOVTRAP system. Illumination with blue light converts the LOV domain into the light state and displaces the dark-state binding Zdk tag and associated molecule (here, RIAM) from the mitochondria to enable adhesion binding. (**B**) Imaging of cells with a blue laser immediately displaces RIAM from a mitochondria localization (RIAM channel, magenta inset), leading to increased adhesion binding of RIAM and reduced adhesion binding of DLC1 (green inset and time series on right); the dynamics is changed after FAK inhibition. (**C** and **D**) Average time intensity curves of adhesions from 15 cells from three independent repeats for control and FAK inhibition, respectively. (**E** to **H**) Adhesion intensities (normalized to whole cell intensities) at the time of peak adhesion enrichment for RIAM, displayed as box plots, and changes for each cell from three independent repeats and >5 cells quantified per repeat. (**I**) Time for RIAM to reach peak intensity at adhesion. (**J**) Time for DLC1 to return to initial intensity before stimulation. *P < 0.05; **P < 0.01; ***P < 0.001; ****P < 0.0001, paired *t* test.

**Fig. 6.**
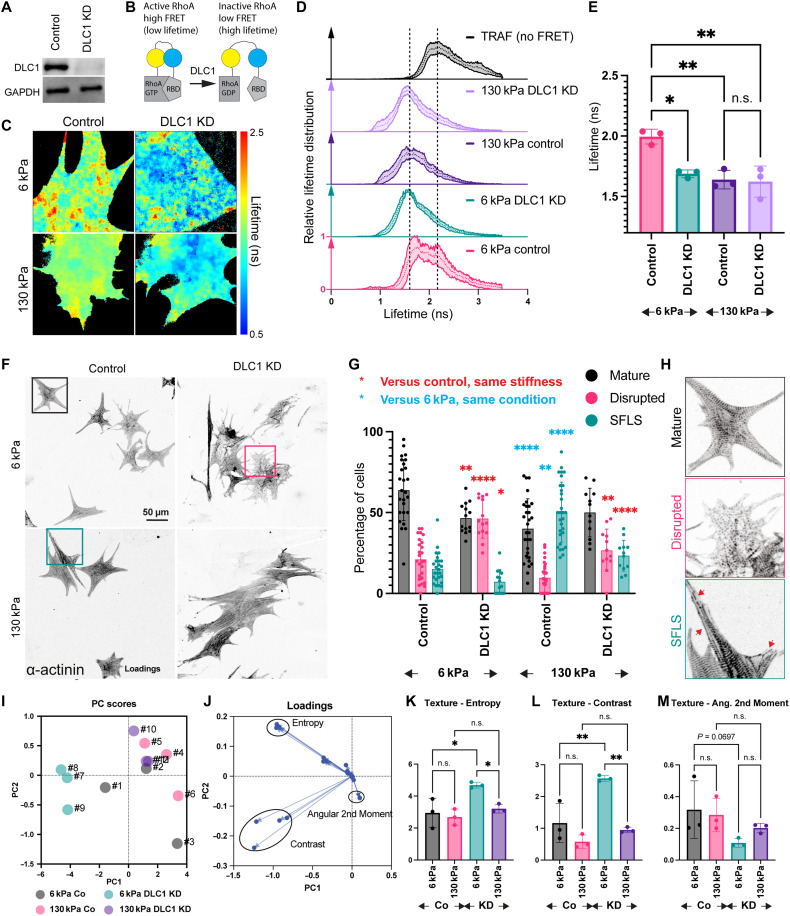
Loss of DLC1 leads to a stiffness-dependent reduction in RhoA activity and sarcomeric organization. (**A**) DLC1 siRNA validation. (**B**) Schematic of RhoA biosensor (*44*). RBD, Rho binding domain. (**C**) Example fluorescence lifetime images from three independent, biological repeats and nine cells each; (**D**) distribution of lifetimes per pixel; TRAF, Tumor Necrosis Factor (TNF) receptor-associated factors domain, which is inserted between the FRET pair as no FRET control. (**E**) peak lifetime after log-normal fit, averaged per repeat; (**F**) α-actinin staining in neonatal rat cardiomyocytes indicates a loss of sarcomeres after DLC1 knockdown (KD), quantified in (**G**) from three independent, biological repeats with 12 to 30 images quantified per condition. Examples for the different categories shown in (**H**), which are zooms into the areas indicated by boxes in (F). SFLS, stress-fiber like structures. (**I**) Principal component (PC) analysis of 52 “texture” measurements (i.e., 13 types with four measurement scales each) shows separation of 6 kPa DLC1 knockdown samples (numbers 7 to 9) from other groups. (**J**) Loading plot indicated Entropy, Contrast, and Angular 2nd Moment (multiple points each for the output from different measurement scales) to have the largest loading (i.e., correlation) with the principal components. (**K** to **M**) Texture measurements (mean per repeat from three independent repeats) for Entropy (K), Contrast (L), and Angular 2nd Moment (M), which had the largest loadings for the principal component analysis (see also fig. S8B and Materials and Methods for further explanation of measurements). *P < 0.05; **P < 0.01; ***P < 0.001; ****P < 0.0001, one-way ANOVA with Dunnett correction for multiple comparisons (E and K to M) or two-way ANOVA with Tukey correction for multiple comparisons (G).

To further test for the competition between DLC1 and RIAM for talin and its alteration through phosphorylation in cells, we used an optogenetic approach. The LOVTRAP system (*43*) uses a small protein tag (Zdark, or Zdk) that has been engineered to selectively bind the Light-oxygen-voltage-sensing domain (LOV2) photosensor domain from the *Avena sativa* phototropin 1 protein in the dark state only. The LOV2 domain is linked to a fragment of the Translocase Of Outer Mitochondrial Membrane 20 (TOM20) mitochondrial anchoring sequence and hence sequesters any Zdk-tagged proteins to the mitochondria in the dark state. Upon illumination with blue light, the Zdk-tagged protein is rapidly released from the mitochondria and is free to interact with its typical interaction partners. Therefore, this system is ideally suited to investigate the direct competition between RIAM and DLC1 at the talin R8 domain (schematic in Fig. 5A). Because of the technically challenging nature, we performed the experiment in the C2C12 muscle cell line and on glass coverslips. As expected, when cells were transfected with a combination of DLC1–green fluorescent protein (GFP), RIAM-mCherry-Zdk, and TOM20-LOV, cells kept under dark conditions showed that RIAM was localized to the mitochondria (Fig. 5B, magenta inset), which was further confirmed by colabeling with the MitoSpy mitochondria tracker (fig. S7A). Illumination with blue light immediately dislocated RIAM from the mitochondria and resulted in an increased adhesion localization (Fig. 5, C, E, and I; time to peak: 5.0 ± 0.4 s). In contrast, adhesion-localized DLC1 was reduced (Fig. 5, C and G), confirming a competition of RIAM with DLC1 for adhesion localization. After an initial phase of RIAM enrichment, DLC1 returned to prestimulation levels (Fig. 5C). No change in DLC1 localization was observed when mCherry-Zdk was transfected as a control, instead of RIAM-mCherry-Zdk (fig. S7, B and C). Treatment with the FAK inhibitor did not affect the adhesion localization per se (Fig. 5, D, F, and H) but resulted in a change in the dynamics, especially a delayed enrichment of RIAM at the adhesions (Fig. 5I; time to peak: 7.8 ± 0.5 s) and delayed return of displaced DLC1 to the adhesions (Fig. 5J; 14.2 ± 0.7 s after FAK inhibition compared to 10.8 ± 0.5 s for the control), overall agreeing with a shift in the affinities toward stronger RIAM and reduced DLC1 binding to talin. Endogenous RIAM localized to adhesions at 130 kPa, but no clear RIAM staining was detected at cardiomyocyte adhesions at 6 kPa (fig. S8). Consistent with a competition in cells, a knockdown of DLC1 in cardiomyocytes using small interfering RNA (siRNA) (Fig. 6A) resulted in increased binding of endogenous RIAM to adhesions at 6 kPa, but no additional adhesion enrichment was observed after DLC1 knockdown at 130 kPa (fig. S8).

### Loss of DLC1 leads to stiffness-dependent changes in RhoA activity and sarcomeric structure

Because DLC1 appears to be the dominant RhoGAP in cardiomyocyte adhesions, we next wanted to test whether it was involved in regulating RhoA activity. For this, we knocked down DLC1 in NRCs and measured the RhoA activity by fluorescence lifetime imaging of a RhoA biosensor. This single-chain biosensor is based on the ability of the Rho binding domain to interact with active guanosine 5′-triphosphate (GTP)–RhoA but not with inactive guanosine diphosphate (GDP)–RhoA (*44*). The interaction brings the fluorophores [cyan fluorescent protein as fluorescence resonance energy transfer (FRET) donor and yellow fluorescent protein as FRET acceptor] in close proximity and thus results in a shortening of the fluorescence lifetime of the donor in the active GTP-bound state (Fig. 6B). Consistent with a higher amount of DLC1-talin interaction at 6 kPa, we found a longer lifetime, i.e., reduced activity of RhoA in cardiomyocytes cultured at 6 kPa, compared to 130 kPa. Moreover, the knockdown of DLC1 increased the RhoA activity (i.e., decreased the lifetime) at 6 kPa to levels comparable to what was detected at 130 kPa, while the knockdown of DLC1 in cardiomyocytes on 130-kPa PDMS had no additional effect on the lifetime (Fig. 6, C to E). When we assessed the effect of the knockdown on myofibrillar structures, using α-actinin as a marker for sarcomeres, we found a reduction in cells displaying fully mature sarcomeres at 6 kPa and an increase of cells displaying myofibrillar disarray (Fig. 6, F to M). This was also evidenced by quantifying the texture measurements using CellProfiler: A principal component analysis indicated a clear separation of the 6-kPa DLC1 knockdown conditions, whereby measurements for entropy (evaluating the image complexity), contrast (evaluating local variation), and angular second moment (evaluating homogeneity) correlated strongest with the principal components (Fig. 6J and fig. S9 for evaluation of the measurements on a set of idealized cells). Consistently, we detected increased entropy and contrast after DLC1 knockdown at 6 kPa. The angular second moment was reduced, although this was not significant. Cardiomyocytes at 130 kPa displayed an overall larger proportion of cells containing long stress fiber–like structures even under the control condition (as previously reported) (*16*). However, this was shifted toward disrupted sarcomeres after DLC1 knockdown, although only minor, nonsignificant differences were detected with the texture measurements.

**Fig. 7.**
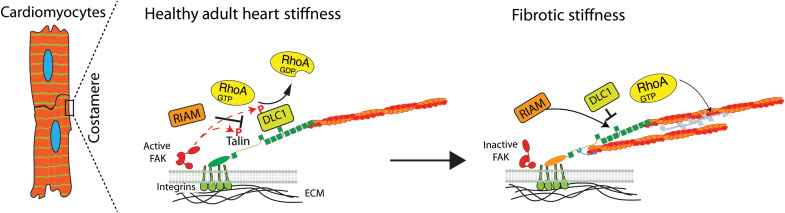
Model of stiffness-dependent competition of talin interactome. Stiffness-dependent integrin signaling leads to altered levels of FAK activation. On a healthy stiffness, DLC1 has a higher affinity for binding to the talin R8 domain compared to RIAM, possibly due to direct phosphorylation through FAK (*48*), and, thus, can modify the level of RhoA activity. In addition, FAK is predicted to phosphorylate talin, and the phosphorylation might further contribute to the regulation (*64*). On a fibrotic stiffness, lower FAK activity reduces DLC1 phosphorylation, and DLC1 is outcompeted by RIAM for talin R8 binding. Because of the importance of DLC1 for fine-tuning the levels of RhoA activity, this affects the cardiomyocyte maturity and disease progression.

Notably, adenoviral driven overexpression of DLC1 also reduced the maturity of the cardiomyocytes but, in this case, led to an increase in the number of cells with excessive stress fiber–like structures, reaching a level that was otherwise seen on fibrotic stiffness (130 kPa, with and without overexpression of DLC1; fig. S10). Overall, this suggests that mechanical changes (*1*) in the talin interactome lead to alterations in DLC1 activity, and the level of DLC1 at adhesions finely tunes the activity of RhoA in a stiffness-dependent way to affect sarcomeric integrity, cardiomyocyte function, and pathological cardiac remodeling (Fig. 7) (*25*, *30*).

## DISCUSSION

Mechanical signaling has been well described as a critical factor influencing health and disease (*45*). Integrin adhesions play an integral role in the mechanical sensing of ECM stiffness. Cell type–specific adhesion composition allows adaptation to particular mechanical environments, which is coupled to the cell phenotypic regulation through mechanotransduction pathways. Rho GTPases are critical components of the mechanotransduction pathways. They act as molecular switches and, among other functions, regulate contractile force generation or the assembly of the cytoskeleton (*46*, *47*). GEFs and GAPs allow the regulation of the activity of GTPases in a spatiotemporal and cell type–specific way (*35*). Here, we find that DLC1 is the key RhoGAP in cardiomyocyte adhesions and interacts with talin in a stiffness-dependent way. This interaction is regulated through changing affinities of RIAM and DLC1 for the shared binding site on talin. Moreover, SFK and FAK inhibition simultaneously reduced DLC1 and increased RIAM binding to talin in co-IPs and FAK inhibition altered the dynamics in FRAP experiments accordingly. Matching with this, Western blotting further showed elevated levels of active phosphorylated FAK in NRCs at 6 kPa, consistent with higher levels of DLC1-talin binding, and our data indicate that the direct competition between DLC1 and RIAM for talin binding depends on FAK phosphorylation, as was also evidenced by our optogenetic approach. The competition for talin binding is further supported by the in vitro competition experiments (Fig. 4), and since the inhibitor studies investigated relative changes due to phosphorylation, these experiments (LOVTRAP, co-IP, and FRAP) demonstrate a clear role for FAK in the regulation of the interaction. Consistent with our findings, a previous study indicated that FAK can activate DLC1 in an indirect mechanism (i.e., by inhibiting the interaction between DLC1 and the phosphatase PP2A) (*48*), and FAK and DLC1 were previously found to bind to each other through a direct interaction between the LD-like motif on DLC1 and the FAK FAT domain (*49*).

Our finding of stiffness-dependent activation of FAK adds to a long list of previous studies of FAK phosphorylation. Generally, these studies that are performed using a variety of different cell types find an increase in FAK activation, from soft to stiff, including in Madin-Darby canine kidney cells (increase from ~50 Pa to 1 kPa with a saturation at ~750 Pa) (*50*), human umbilical vein endothelial cells (increase from 2.5 to 10 kPa) (*51*), mammary epithelial cells [increase from 6 to 18 kPa in two-dimensional (2D) culture and from 25 to 44 kPa in 3D] (*52*), MHCC97H hepatocellular carcinoma cells (increase from 6 to 16 kPa) (*53*), serum-starved mouse embryonic fibroblasts (MEFs), and vascular smooth muscle cells after serum stimulation (increase from 2 to 4 kPa to 20 to 25 kPa) (*54*). In contrast, an investigation of MEFs on micropillars found no difference in average levels of total FAK, or pY397 FAK between 5 and 14 kPa, although it reported a dependence of FAK activation on intracellular force generation or external force application through magnetic tweezers (*55*).

Overall, our finding of increased FAK pY397 levels at 6 versus 1 kPa fits well with previous studies, although, to our knowledge, the biphasic behavior of FAK activity, with down-regulation at 130 kPa, has not been reported before. However, biphasic stiffness sensing is in agreement with reports of other mechanosensitive processes, including epigenetic states (*56*), cell migration (*57*), and T cell activation (*58*), and future studies will need to investigate whether this is a general behavior or specific for cardiomyocytes.

The role of SFK, on the other hand, is less clear. Previous studies mostly investigated Src in other cell types (especially immune cells), whereas in cardiomyocytes, Src is expressed only at low levels, compared to Fyn and Yes (which are both inhibited by PP2). Moreover, in our hands, pSFK bands on Western blots did not overlap with Src (fig. S6). A previous study by Tripathi *et al*. (*41*) found Src inhibition in human embryonic kidney–293 cells with saractinib (which inhibits both Src and Abl), but not FAK inhibition–reduced RhoA activity. They identified that Src phosphorylation of DLC1 Y701 reduced the GAP activity and led to higher RhoA activity. The study further showed that Yes and Fyn equally phosphorylate DLC1 but less efficiently. However, they did not investigate the specific phosphorylation sites. Since other Src phosphorylation sites were identified, including Y451, which was affecting tensin binding but not GAP activity, it remains unclear whether Fyn and Yes can indeed regulate RhoA via DLC1.

In the case of RIAM, a separate study by Chang *et al.* (*59*) indicated that Y45 might be involved in RIAM activation and consequently found increased plasma membrane recruitment after mutation of this residue in Jurkat T cells. Likewise, the inhibition of FAK reduced plasma membrane translocation in these cells. However, RIAM binding to the talin R8 domain might be context dependent and missing in Jurkat T cells. Hence, it might not have been detected in this assay, e.g., because of a stronger presence of DLC1 or paxillin at the binding site on R8. Future studies will need to identify how FAK is acting on the different binding partners, i.e., which sites of which molecules will be phosphorylated and how this affects the complex relationship in their interactions with talin.

Nevertheless, we identify here that the competitive talin interactions affect downstream mechanosignaling. DLC1 knockdown increases RhoA activity and, at the same time, leads to myofibrillar disruptions. On the other hand, an increase in DLC1 expression is linked with the formation of long stress fiber–like structures. This suggests that RhoA activity in cardiomyocytes needs to be finely tuned, as supported by the literature pointing to both benefits and detrimental effects of RhoA in cardiovascular health and disease (*30*): RhoA is up-regulated in early heart development (*28*), but RhoA overexpression in the adult heart results in dilated cardiomyopathy (albeit only at high, not at moderate, overexpression) (*29*, *60*). Then again, cardiomyocyte-specific knockout of RhoA resulted in an accelerated and more severe dilated cardiomyopathy after chronic pressure overload (*25*).

DLC1 deletion is embryonic lethal and linked to heart development defects (*61*, *62*), and several DLC1 variants have been identified in a Chinese cohort for sporadic congenital heart disease (*63*). This indicates an important role for the DLC1-RhoA axis in heart development and disease. Coupling DLC1 activation and localization to the central mechanosensitive scaffold protein talin at sites of adhesion provides a way to regulate this axis in response to a changing mechanical environment. As the stiffness changes, the levels of DLC1 at adhesions are altered. This fits with altered talin conformations changing the accessibility and localization of the binding site for DLC1. However, the preservation of the stiffness-dependent interactions with talin after removal from the mechanical environment further indicates mechanosensitive biochemical imprinting where these force-dependent states and interactions are being stabilized. Our work highlights the role of tyrosine kinases in this process. Tyrosine phosphorylation of talin is reported in PhosphoSitePlus as found in hundreds of high-throughput screens, and FAK and SFKs are predicted kinases involved in this process (*64*). Therefore, it is tempting to speculate that the effects of FAK reported in this work are, at least in part, due to alterations in the phosphorylation of talin itself. The pathway we outline here only partially reflects the complexity of the talin mechanical interactions in the cardiomyocytes.

In addition, other posttranslational modifications of talin might further affect force transduction and sensing. For instance, methylation of R13 by Ezh2 has been shown to disrupt actin binding (*65*) and, hence, force transduction. Similarly, cyclin-dependent kinase 1 (CDK1) was described to bind to the R8 talin domain, and CDK1 phosphorylation of a serine residue (S1589) in the linker between R7 and R8 reduces the force needed for the unfolding of the R8 domain (*66*).

Limitations of our current study include a relatively simplified experimental model to facilitate discovery of the processes at play, mostly using in vitro experiments, albeit in situ PLAs also confirmed our findings in animal models. While the DLC1 displacement using RIAM LOVTRAP release worked well, we were not able to reliably measure RIAM displacement using a DLC1 LOVTRAP release from the mitochondria, because of residual adhesion localization of DLC1 even in the dark state. In addition, because of the complexity of the experiments, C2C12 cells were used instead of cardiomyocytes, and experiments were performed on healthy adult heart stiffness only, rather than the whole stiffness range.

Last, paxillin has also been previously identified as a costameric protein in the heart. Paxillin was involved in the regulation of cardiac contractility in zebrafish embryos (*39*), in agreement with the strong association with talin at embryonic stiffness in our study. Notably, while paxillin protein levels were seemingly unaltered in heart disease (*40*), genome-wide association studies nevertheless indicate links with “left ventricle wall thickness,” “left ventricular function,” “hypertrophic cardiomyopathy,” and other traits related to heart disease [National Human Genome Research Institute–European Bioinformatics Institute genome-wide association study (NHGRI-EBI GWAS) Catalog (*67*)]. In contrast, to our knowledge, RIAM has not been studied in cardiomyocytes in detail. However, the increased expression in heart disease as well as interaction with talin at fibrotic stiffnesses could indicate a potential involvement in the mechanisms of heart disease as well. Together, this warrants a deeper investigation of paxillin and RIAM into a potential involvement in heart development and disease.

For now, however, we focused on DLC1, and our results show that mechanical sensing and activation of integrin-related kinase pathways, including FAK, alter the talin interactome. This leads to an imprinting of the mechanical information to determine RhoA activity and to regulate the cardiomyocyte phenotype and function.

## MATERIALS AND METHODS

### Antibodies and reagents

The following primary antibodies were used in the study: talin (rabbit; Abcam, ab71333), talin (mouse; Bio-Rad, MC4770GA), α-actinin (Sigma-Aldrich, A7811), vinculin (Sigma-Aldrich, V9131), DLC1 (rabbit; Novus Biologicals, NBP1-88824PEP), DLC1 (mouse; BD Biosciences, 612020), paxillin (BD Biosciences, 610052), RIAM (Abcam, ab92537), glyceraldehyde-3-phosphate dehydrogenase (GAPDH) (Abcam, ab8245, and Cell Signaling Technology, 97166), phospho-SRC family (Tyr^416^; Cell Signaling Technology, 2101), and total Src (Merck, 05184). The following reagents were used at the listed concentrations: BIS I (500 nM; Sigma-Aldrich), PP2 (10 μM; Cayman Chemical), Y27632 (10 μM; Enzo Life Sciences), and FAK inhibitor 14 (1 μg/ml; Cayman Chemical).

pTriEx-NTOM20-LOV2 (Addgene plasmid no. 81009) and pTriEx-mCherry-Zdk1 (Addgene plasmid no. 81057) were a gift from K. Hahn (*43*). The RhoA-FRET biosensor was a gift from O. Pertz (*44*). Adenoviral constructs for DLC1-GFP (based on the sequence NM_001348081.2), RIAM-GFP (based on the sequence NM_019043.4), and RIAM-mCherry-Zdk1 (based on pTriEx-mCherry-Zdk1 and NM_019043.4) were generated by VectorBuilder.

### Human tissues

Surplus human tissue from patients undergoing surgery was snap-frozen in liquid nitrogen. Specifically, leftover biological materials (e.g., cardiac tissue and biological fluids) were collected during routine clinical procedures and stored under protocol BB-CCH at the UOC (Unità Operativa Complessa) of Cardiac Surgery, Azienda Ospedaliera Universitaria Integrata Verona (see also the “Ethics Statement” section below). Patients were informed that participation was voluntary and that they could withdraw consent at any time without consequences. Samples and associated clinical data were de-identified and managed under strict confidentiality, with appropriate data protection measures in place. All participants provided written informed consent for the collection, storage, and future use of their biological samples and associated data. Transfer of samples or data to external collaborators (including institutions outside the EU) was permitted only in anonymous form and in compliance with General Data Protection Regulation and institutional policies. For patient characteristics, see table S1. Samples from left ventricular assist device implantation and explanted hearts were in end-stage heart failure [ejection fraction (EF) of <40%], while aortic stenosis samples (apart from one exception) had preserved cardiac function (EF of >50%).

### Mouse tissues

MLP knockout mice, a genetic model for heart failure, and WT controls (C57BL/6J, male and female) were euthanized at 6 months of age, and hearts were snap-frozen for cryosections. Samples for Western blotting were from MLP knockout mice and WT controls (C57BL/6J, 3× male and 3× female), euthanized at 10 weeks of age.

### PDMS substrates

PDMS substrates were prepared, by mixing Sylgard 527 with Sylgard 184 as described previously (*68*). Sylgard 184, Sylgard 527, or mixtures at the ratios of 1:5, 1:10, and 1:20 were spin-coated with a 150i spin processor onto coverslips at 1000 rpm for 100 s and curing at 70°C for 12 hours. Before spin coating, Sylgard 527 was precured at 70°C for 30 min with intermitted mixing to achieve a comparable viscosity to the Sylgard 184 mixture. The stiffness of the mixtures was measured by rheology as described previously (*16*).

### Cardiomyocyte isolation, cell culture, and siRNA

Neonatal rat cardiomyocytes were isolated from newborn Wistar rats (male and female), using the sequential digestion method as described previously (*69*). Briefly, hearts were dissected into ice-cold Artificial Dissection Solution (ADS) buffer (116 mM NaCl, 20 mM Hepes, 0.8 mM NaH_2_PO_4_, 5.6 mM glucose, 5.4 mM KCl, and 0.8 mM MgSO_4_). After the hearts settled down to the bottom, they were washed once with ADS buffer. ADS buffer was then removed, and hearts were incubated with 5 ml of enzyme solution (ES) in ADS [collagenase (246 U) and pancreatin (0.6 mg/ml)] for 5 min at 37°C under vigorous shaking. The supernatant was discarded. This step was followed by five to six digests, until hearts were completely digested. Each time, 5 ml of fresh ES was added to the hearts, and these were incubated for 15 min at 37°C, under shaking. Hearts were pipetted up and down 30 times using a Pasteur pipette. After settling down, the supernatant was transferred into the plating medium [65% Dulbecco’s modified Eagle’s medium (DMEM), 17% M199, 10% horse serum, 5% fetal bovine serum (FBS), 2% GlutaMAX, and 1% penicillin/streptamycin (P/S)]. Two digests each were combined in one tube with 20 ml of plating medium, then cleared through a 100-μm cell strainer and spun down at 1200 rpm for 5 min at room temperature, before being resuspended in 10 ml of plating medium. Cells were pooled together and preplated for 90 min to enrich the cardiomyocytes. Cardiomyocytes were then plated onto the respective substrates as indicated in the text or figures. Medium was changed the next day to maintenance medium (77% DMEM, 18% M199, 2% horse serum, 2% GlutaMAX, and 1% P/S) or serum starvation medium (as above, but excluding the horse serum).

DLC1 knockdown was performed using 1 μM DLC1 Accell siRNA SMARTpool (mix of four siRNA targeting the DLC1 sequence GUAUGAGCAUCUAUGACAA, CUCCUGUCCAGAUAUUUGA, CUAUUAUACCUGGUUUAGC, and GUUUUAGCAUGAAAGGUCA) (no. E-088843-01-0010 from Horizon), which required no transfection reagent for delivery. The scrambled siRNA was a nontargeting siRNA (Accell no. D-001910-01-05). Cells were incubated for 48 hours after transfection. C2C12 cells were obtained from American Type Culture Collection (CRL-1772) and cultured in high-glucose DMEM with 4 mM l-glutamine and 1 mM sodium pyruvate, substituted with 10% FBS and 1% P/S.

### Western blotting and co-IP

Snap-frozen cardiac samples [either from human patients at the time of surgery or from mice culled by cervical dislocation, after quick rinse in phosphate-buffered saline (PBS)] were powdered with a liquid nitrogen–cooled BioPulverizer 100 (BioSpec Products). Samples were mixed with 2× SDS sample buffer (125 mM tris-HCl at pH 6.8, 200 mM dithiothreitol, 4% SDS, 12% glycerol, and 0.05% bromophenol blue, using 200 μl of 2× SDS sample buffer per 100 μg of tissue), heated at 65°C for 15 min and then sonicated and subjected to Western blotting.

Neonatal rat cardiomyocyte protein lysates were extracted using radioimmunoprecipitation assay buffer with phosphatase and protease inhibitors. Lysates for co-IP were extracted using Pierce IP Lysis Buffer (87788). Co-IPs were performed using the SureBeads Protein A magnetic beads from Bio-Rad, washed with dilution buffer (10 mM tris, 150 mM NaCl, and 0.5 mM EDTA at pH 7.4) and coated with the talin antibody (Abcam, ab71333; 5 μg/200 ml). The protein lysates were added to the beads for 1 hour at room temperature before being washed with washing buffer (10 mM tris, 150 mM NaCl, 0.5 mM EDTA, and 0.05% Triton X-100), eluted in 20 mM glycine at pH 2.0 for 5 min at room temperature, and neutralized with 1 M tris at pH 10.4. The protein dephosphorylation was performed on total WT and MLP knockout heart lysates using recombinant shrimp alkaline phosphatase (rSAP) (New England Biolabs, M0371S; 1 U of rSAP/10 mg of protein). The protein lysates containing phosphatases, but omitting phosphatase inhibitors, were incubated for 30 min at 37°C, before subjecting the samples to the co-IP as described above.

Samples were separated on Mini-PROTEAN TGX Precast Gels (4 to 15%, Bio-Rad), and loading was adjusted based on the intensity of bands after Coomassie stain. For Western blotting, samples were separated in gels and transferred onto nitrocellulose or polyvinylidene difluoride membranes. The transfer was verified by Ponceau S stain (0.1% Ponceau and 0.5% acetic acid, Merck). The membranes were cut depending on the molecular weights of the desired proteins and rinsed in 0.1% tris-buffered saline–Tween. A 5% milk in tris-buffered saline–Tween blocking buffer was then applied to the membrane for 1 hour at room temperature. Primary antibodies were incubated at 4°C overnight or for 1 hour at room temperature for GAPDH. Horseradish peroxidase–conjugated secondary antibodies were incubated for 1 hour at room temperature.

### Immunofluorescence

Hearts were covered with optimal cutting temperature for cryosectioning on a cryostat (Leica Biosystems). Frozen sections were fixed in ice-cold acetone at −20°C for 5 min before rehydration in PBS and blocking for 30 min with 5% goat serum in PBS. Neonatal rat cardiomyocytes were fixed using 4% paraformaldehyde for 10 min at room temperature, permeabilized with 0.2% Triton X-100 for 5 min at room temperature and blocked for 1 hour at room temperature with 5% goat serum. Immunostaining of heart sections and neonatal rat cardiomyocytes on PDMS was performed by incubating the samples with primary antibodies at 4°C overnight in a humidified chamber. On the next day, after three 5-min washings with PBS, samples were incubated for 1 hour with secondary antibodies, phalloidin and 4′,6-diamidino-2-phenylindole (DAPI), at room temperature. Samples were washed three times before mounting using Fluoromount Mounting Medium (Thermo Fisher Scientific).

### Microscopy

Optogenetic and FRAP experiments were performed using a Nikon CSU-W1 SoRA Spinning Disk confocal microscope with two Photometrics Prime BSI cameras and equipped with an environmental chamber for CO_2_ and temperature control, using a 60× oil Lambda Apochromat objective and after transfection of the neonatal rat cardiomyocytes for 48 hours. For FRAP, five frames at 3 fps were acquired to record the baseline, before laser stimulation of 3-μm circular regions of interest (ROIs) on focal adhesion sites at 10% laser power for 200 ms. Recovery after photobleaching was observed for 3 min with acquisition every 3 s. For optogenetic experiments, cells were located and then left for 2 min in the dark to restore the mitochondrial localization of RIAM-mCherry-Zdk or mCherry-Zdk. Images were acquired for 30 s at 1 fps using both 488- and 568-nm laser lines, whereby the first 488-nm exposure was sufficient to convert LOV into the light state and displace RIAM-mCherry-Zdk or mCherry-Zdk from the mitochondria.

Mitochondria localization was confirmed by labeling the cells with the MitoSpy NIR DiIC1 mitochondrial localization probe (BioLegend), according to the manufacturer’s instructions. FRET acquisitions of the single-chain RhoA-FRET biosensor were performed on a custom-built multibeam confocal fluorescence lifetime imaging microscopy system, as described previously (*70*).

### In situ proximity ligation

Heart slices were permeabilized using 0.2% Triton X-100 and blocked with the Duolink blocking solution for 1 hour at 37°C. The antibodies were used in the following combinations: RIAM (rabbit)/talin 1 (mouse) and DLC1 (mouse)/talin 1 (rabbit). All Duolink PLA reagents were obtained from Sigma-Aldrich. Antibodies were diluted in Duolink antibody diluent buffer and washed with washing buffer A [NaCl (8.8 g/liter), tris base (1.2 g/liter), and 0.05% Tween-20 in ddH_2_O]. The PLA probes PLUS and MINUS, containing an affinity-purified, oligonucleotide-conjugated antibody against either mouse or rabbit, were in Duolink antibody diluent buffer, incubated on heart slices for 1 hour at room temperature and washed with washing buffer A. Ligation solution (2.5% ligase and 20% ligation stock solution in ddH_2_O) was added to each sample, incubated for 30 min at 37°C and washed with washing buffer A. The amplification step was performed by adding the amplification solution (1.25% polymerase and 20% amplification stock solution in ddH_2_O) to the heart slices, incubated for 100 min at 37°C, and washed with washing buffer B [NaCl (5.84 g/liter), tris base (4.24 g/liter), and tris-HCl (26 g/liter), at pH 7.5]. Heart samples were counterstained with phalloidin and DAPI before mounting and imaging.

### Nanoindentation

Nanoindentation experiments were performed using an Optics11 Chiaro nanoindenter attached to a Leica DMi8 microscope as described previously (*71*). Measurements were performed on 20-μm-thick tissue sections with an *R* = 50 μm, *k* = 0.5 N/m probe, and 2-μm indentations in the matrix scan mode. Young’s moduli were calculated using the Optics11 data viewer software, using the Hertz model. Images of the section with the probe in contact at the start and end positions were taken for alignment of the measurements with the bright-field image.

### Protein expression

Talin R7R8 was expressed and purified from *Escherichia coli* BL21 (DE3) Star cells transformed with pet151D-topo plasmid containing mouse talin 1 R7R8. A cryostock of transformed cells was used to inoculate 100 ml of LB/ampicillin (100 μg/ml) and grown overnight (18 hours) at 37°C with 220-rpm shaking. For protein expression, 5 ml of the overnight culture was added to 750 ml of LB/ampicillin (100 μg/ml) in 2-liter baffled flasks and then incubated at 37°C with 180-rpm shaking until optical density (OD) = 0.40 was reached. The temperature was reduced to 20°C, and the cultures were allowed to grow for 30 min to OD ~0.7. The cultures were induced with 0.4 μM isopropyl-β-D-thiogalactopyranoside (0.4 μM) and grown overnight (20 hours) at 20°C. Cells were pelleted with 6000*g* centrifugation at 4°C for 10 min, and the supernatant was discarded. Whole cell pellets were resuspended with 5 ml of resuspension buffer (50 mM tris at pH 8, 250 mM NaCl, and 10% w/v glycerol) per gram of wet pellet and stored at −20°C.

Frozen cell pellets were thawed and stirred with the addition of 1 mM Tris(2-carboxyethyl)phosphine (TCEP), 1 mM phenylmethylsulfonyl fluoride, and Triton X-100 (0.2% v/v) for 5 min. The cells were sonicated to disrupt the membranes using pulses of 3 s on (35% amplitude) and 7 s off, for 4 min total sonication time. Then, cellular debris were pelleted at 40,000*g* at 4°C for 45 min. The lysate supernatant was sterile-filtered through a 0.22-μm syringe filter and loaded onto a 5-ml nickel column (HisTrap) using an AKTA start, washed with 50 mM tris (pH 8), 600 mM NaCl, 30 mM imidazole, 10% w/v glycerol, 0.2% Triton X-100, and 1 mM TCEP, and then washed with 50 mM tris (pH 8) and 250 mM NaCl. The R7R8 protein was eluted in 3-ml fractions using an imidazole gradient increasing from 0 to 175 mM, followed by a step to 300 mM. SDS–polyacrylamide gel electrophoresis (SDS-PAGE) was used to view and select the fractions with the most R7R8 for continued purification. The selected fractions were combined and diluted 1:5 in 20 mM tris (pH 8) to dilute imidazole, loaded onto a 5-ml anion exchange column (HiTrap Q HP), and washed with 20 mM tris (pH 8) and 20 mM NaCl. Protein was eluted into 3-ml fractions using a NaCl gradient from 0 to 450 mM, followed by a step to 750 mM. SDS-PAGE was used to select R7R8 containing fractions with the highest protein concentration. Purified R7R8 was dialyzed against PBS (137 mM NaCl, 27 mM KCl, 100 mM Na_2_HPO_4_, and 18 mM KH_2_PO_4_, at pH 7.4) at 4°C overnight in 10,000 molecular weight cut-off (MWCO) snakeskin dialysis tubing (Thermo Fisher Scientific). After dialysis, R7R8 was concentrated using a 10,000 MWCO concentration filter to 400 μM. This final protein solution was aliquoted, flash frozen in liquid nitrogen, and stored at −20°C.

### FP assays

DLC1 TBS, RIAM TBS1, and paxillin LD1 peptides with a C-terminal nonnative cysteine were synthesized by GL Biochem (China): DLC1 TBS (465-489C): IFPELDDILYHVKGMQRIVNQWSEKC; RIAM TBS1 (4-30C): SEDIDQMFSTLLGEMDLLTQSLGVDTC; paxillin LD1 (3-23C): MDDLDALLADLESTTSHISKRPC. The binding affinities of DLC1 TBS, RIAM TBS1, and paxillin LD1 peptides to purified R7R8 were determined using an FP assay.

Peptide stock solutions were prepared at 2.5 mM in PBS (with 5% *N*,*N*′-dimethylformamide to solubilize DLC1 TBS). The peptides were coupled to a thiol-reactive fluorescein fluorophore via the terminal cysteine. The uncoupled dye was removed using a PD-10 desalting column. Purified R7R8 in PBS with 1 mM TCEP was serial diluted and added to labeled DLC1 TBS or RIAM TBS1 peptides in PBS with 0.02% Tween-20 to make a final concentration of 0.5 μM labeled peptide and a maximum R7R8 concentration of 160 μM with a final volume of 100 μl along rows of a black 96-well plate in triplicate. Fluorescein FP measurements were recorded (excitation: 485 ± 10 nm, emission: 535 ± 20 nm) using a Hidex plate reader at room temperature. FP values were calculated following $P=\left(F_{∥}-F_{⊥}\right)/\left(F_{∥}+F_{⊥}\right)$ where $F_{∥}$ and $F_{⊥}$ represent the fluorescence intensity parallel and perpendicular to the excitation light plane, respectively. The data were analyzed via GraphPad Prism. *K*_d_ values were calculated by fitting the data to a nonlinear curve using a one-site total binding model.

Competition experiments were performed by first incubating purified R7R8 with DLC1 TBS-F (1 μM) at room temperature in the dark for 20 min. An R7R8 concentration of 6× *K*_d_ (30 μM) was used. This complex was diluted 1:2 by adding 50 μl of this complex to 50 μl of unlabeled RIAM TBS1 or paxillin LD1 peptides at increasing (twofold) concentrations up to a maximum unlabeled peptide concentration of 160 μM. As a negative control, the experiment was performed in the absence of R7R8 where uncomplexed DLC1 TBS-F was added to unlabeled RIAM TBS1 to measure any direct interaction without R7R8. The resulting data were plotted and analyzed via GraphPad Prism. Inhibitory constant (*K*_i_) values were calculated by fitting the data [polarization versus log(peptide)] to a nonlinear curve using a one-site binding model for fitting *K*_i_.

### Quantification and statistical analysis

All experiments were done in at least three independent biological repeats. In the case of neonatal rat cardiomyocytes, this included independent rat litters, cell isolations, and other experimental procedures.

Quantifications were performed using ImageJ and MATLAB. For FRAP experiments, ROIs were drawn around the area of laser stimulation, as well as in a reference location with no bleaching and another one for the background. The background intensity was subtracted from the ROI intensity of the photobleaching spot and divided by the reference intensity. The images preceding laser stimulation were averaged, and data were normalized by subtracting the value of the laser stimulation at T0 from every time point and dividing by the average of the images before stimulation. For quantification of the optogenetic experiments, focal adhesion sites were selected to quantify the intensity after light stimulation. Each value of the time series was divided by the intensity value of the whole cell. For the calculation of time curves, the data were further normalized to the initial value before stimulation.

Western blots were quantified using Image Lab (Bio-Rad) or ImageJ and normalized to GAPDH expression. Protein expression in talin co-IP was normalized on talin expression. The maturity of sarcomeres was quantified using α-actinin expression based on sarcomere morphology and visual presence of stress fiber–like structures or disrupted staining pattern (missing sarcomeres, dot-like Z-discs).

For texture measurements, images were segmented and analyzed with CellProfiler as described previously (*16*). Principal component analysis was performed with GraphPad Prism. The measurements with the greatest loading (i.e., correlation with principal components) were analyzed further and displayed as average per repeat from three independent repeats. The measurements included Angular 2nd Moment, a measure of image homogeneity, whereby a higher value indicates lower intensity variations; Contrast, which measures local variation, whereby a high value indicates a high degree of local variation; and Entropy, a measure for the complexity within an image, whereby a complex image produces a high entropy value. Idealized cells with varying thickness of “myofibrillar” structures and different degrees of disruption were used to evaluate the suitability of the texture measurements. Images were created with Adobe Illustrator to have approximately the same area and separation between sarcomeric striations as the cardiomyocytes in the microscopy images. Images were converted to tiff stacks, together with a second image covering the whole area for segmentation purposes and analyzed in CellProfiler using the same measurement settings as for the cell images.

All statistical tests are indicated in the figure legends. Datasets were tested for normal distribution using the Shapiro-Wilk test. All statistical tests were performed with GraphPad Prism.

### Ethics statement

Animal studies have been performed in accordance with the ethical standards laid down in the 1964 Declaration of Helsinki and its later amendments. Experimental procedures were performed in accordance with the Directive 2010/63/EU and UK Home Office guidelines (project licenses P572C7345 and PDCE16CB0) and approved by the respective institutional ethical review boards.

Ethics for collection of human tissue during surgery for the purposes of establishment of a biobank for left-over biological materials and associated data and experimental use were obtained at the UOC di Cardiochirurgia Verona, protocol code BB-CCH, project 847CESC. Collection of human tissue was conducted in compliance with the ethical standards outlined in the Declaration of Helsinki and the relevant European and Italian regulations concerning biomedical research and data protection (including Legislative Decree 196/2003). All participants provided written informed consent for the collection, storage, and future use of their biological samples and associated data.
