# Supplementary material for: Mechanosensitive biochemical imprinting of the talin interaction with DLC1 regulates RhoA activity and cardiomyocyte remodeling
Source: Sci Adv. 2025 Sep 5;11(36):eadt6083. doi: 10.1126/sciadv.adt6083 (PMC12412656; doi:10.1126/sciadv.adt6083)
Supplement: Supplementary file 1 — Figs. S1 to S10 Table S1 [file sciadv.adt6083_sm.pdf]

Science Advances
AAAS

Supplementary Materials for

Mechanosensitive biochemical imprinting of the talin interaction with DLC1 regulates RhoA activity and cardiomyocyte remodeling

Emilie Marhuenda *et al.*

Corresponding author: Thomas Iskratsch, t.iskratsch@qmul.ac.uk

Sci. Adv. **11**, eadt6083 (2025)
DOI: 10.1126/sciadv.adt6083

This PDF file includes:

Figs. S1 to S10
Table S1

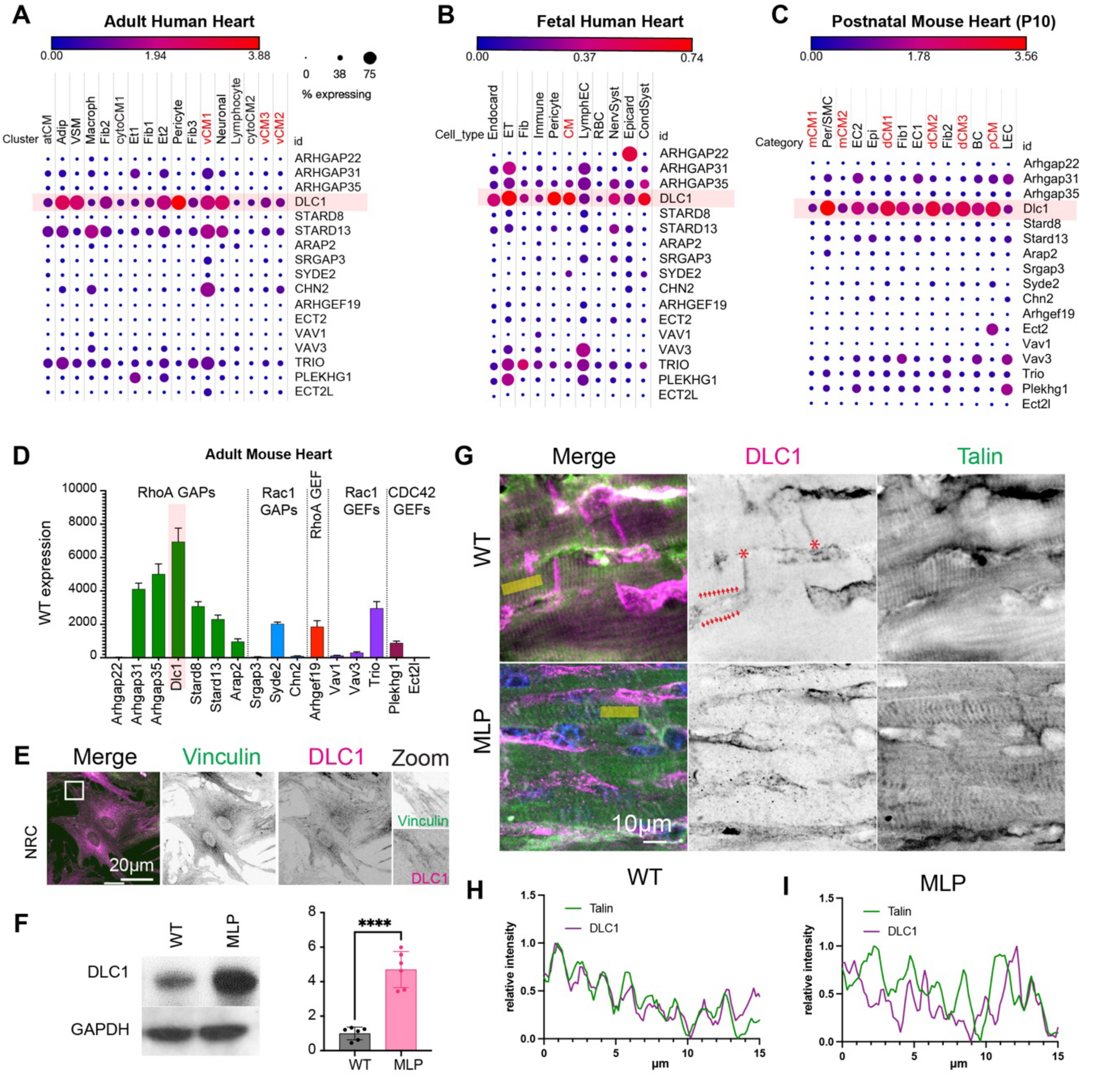

Supplementary Figure S1: DLC1 is a major RhoGAP in cardiomyocytes. Expression of adhesion localised RhoA, Rac1 and CDC42 GAPs and GEFs (as identified by Müller et al *(35)*) was analysed using the Broad Institute Single Cell Portal in **A)** single nuclei RNA-Seq data of adult human hearts (287,269 nuclei from 4 women, 3 men, Age 39-60 years)*(32)*; atCM: atrial cardiomyocytes; Adip: adipocytes; VSM: vascular smooth muscle cells; Fib1,2,3: fibroblast populations; Et1,2: endothelial cell populations; vCM1,2,3: ventricular cardiomyocyte populations; cytoCM1,2: cytoplasmic cardiomyocyte populations. **B)** single cell RNA-Seq data of a normal perinatal human heart (13569 cells, gestational age 83 days)*(33)* **C)** single nuclei RNA-Seq data from postnatal mouse hearts (7760 nuclei from 3 wild type mouse hearts at P10)*(34)*; mCM: mature cardiomyocytes; dCM: developing cardiomyocytes; pCM: proliferating cardiomyocytes; LEC: lymphatic endothelial cells. **D)** The single cell data is further consistent with our bulk sequencing analysis of adult wild type mouse hearts (four wild type mouse hearts)*(36)*; **E)** Immunostaining confirms the adhesion localisation in neonatal rat cardiomyocytes (NRC). **F)** DLC1 expression is increased in the MLP knockout mouse, a model for dilated cardiomyopathy. n=6 hearts for wild-type and the MLP knockout. **** p<0.0001 from unpaired t-test. **G)** Costameric (red arrow heads) and intercalated disc (red asterisks) staining is found in the healthy mouse heart, while the staining is mostly membranous in the MLP heart**. H,I)** profile plots show relative intensities of talin and DLC1 from the line indicated in yellow from panel **(G)**.

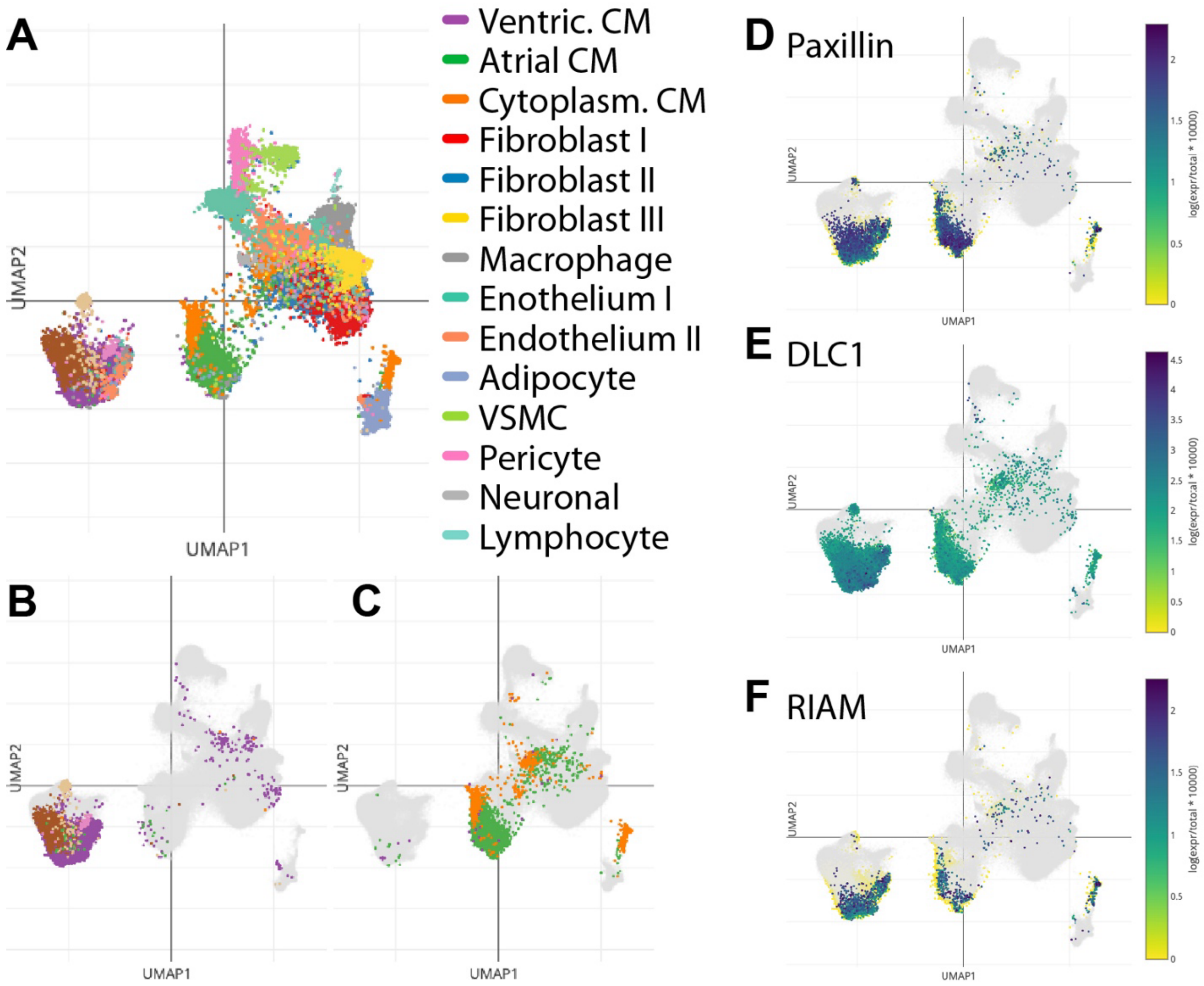

Supplementary Figure S2: Paxillin, DLC1 and RIAM are expressed in cardiomyocytes. Analysis of the scRNA sequencing data from seven human donor hearts (from Tucker et al, Circulation)*(32)* **(A)***,* focusing on cardiomyocyte populations of ventricular **(B)** and atrial **(C)** cells with high quality data (> 1000 UMI detected genes per cell) shows Paxillin **(D)**, DLC1 **(E)** and RIAM **(F)** expressing cells in both ventricular and atrial cardiomyocyte populations.

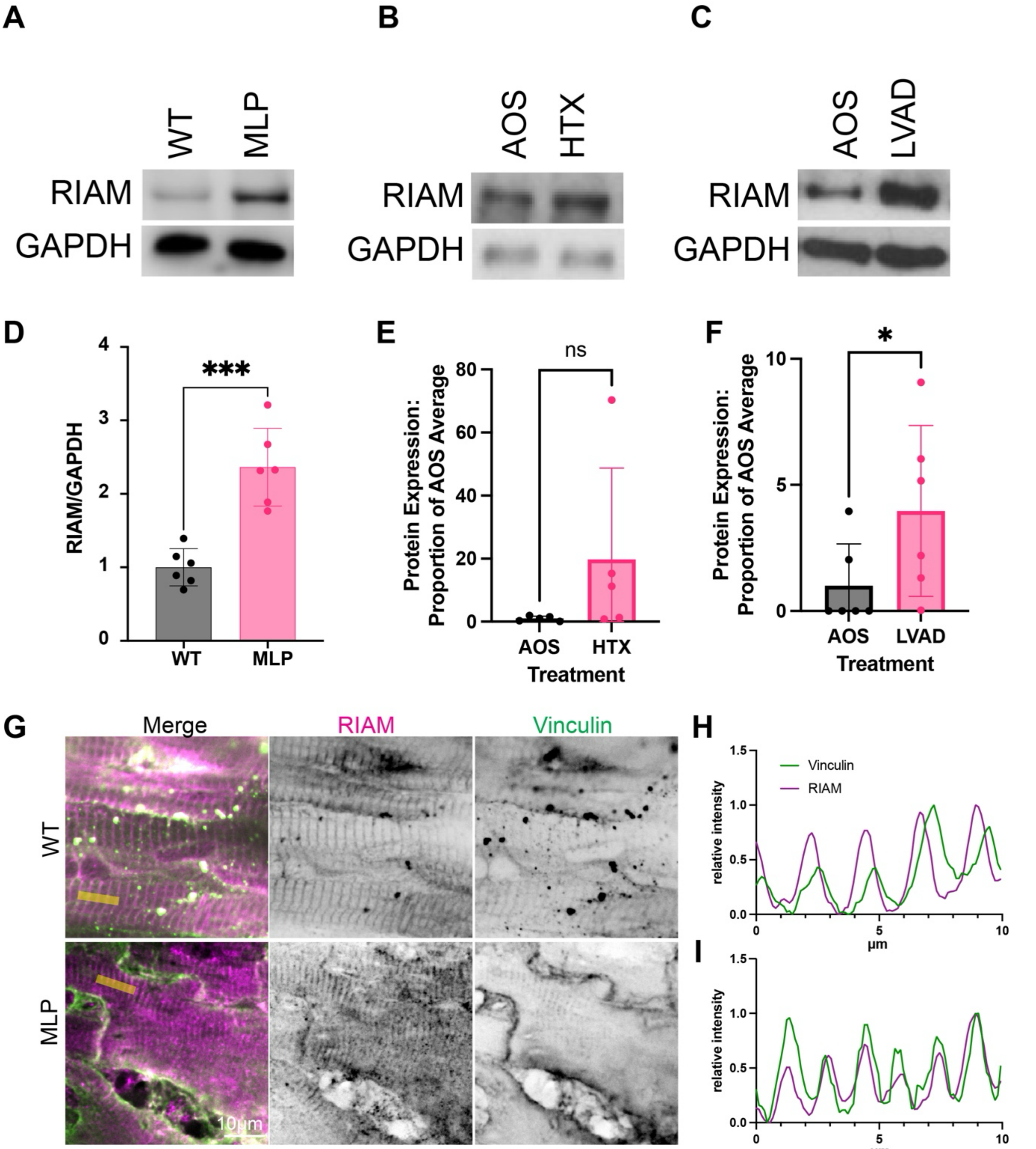

Supplementary Figure S3: RIAM expression is increased in heart disease. A) RIAM is upregulated in MLP knockout hearts; **B)** in failing explanted human hearts at time of explant (HTX) compared to aortic stenosis with preserved ejection fraction as control (AOS, since no healthy human heart samples were available); **C)** in failing hearts at time of implant of left ventricular assistance device (LVAD), compared to AOS; **D-F)** Quantification of **(A)**, **(B)** and **(C)**, respectively; **G)** Immunostaining of mouse heart sections (left ventricle) shows costameric RIAM staining in both wild-type and MLP knockout mouse hearts. **H,I)**: profile plots show relative intensities of vinculin and RIAM from the line indicated in yellow in panel **(G)**. *$p<0.05$, ***$p<0.001$; p-values from unpaired t-test.

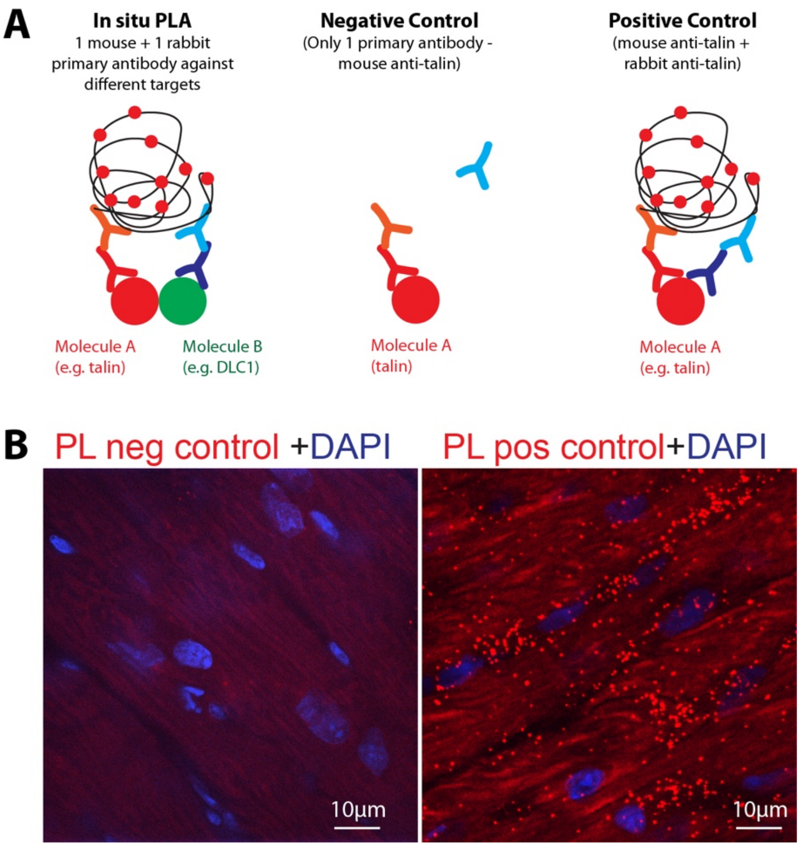

Supplementary Figure S4: Principle of in situ proximity ligation assay and positive and negative controls. A) If in proximity (< 40 nm), PLA probes with connector oligos can hybridise and form a template for DNA amplification. Labelled oligos then hybridise to the complimentary sequences in the amplified DNA, still tethered to the antibodies and can be quantified as discrete spots (PLA signals). **B**) For the negative control, only one primary antibody (mouse anti-talin) was used together with all other reagents (both 2nd antibodies and detection reagents). For the positive control, a mouse anti-talin antibody was combined with a rabbit anti-talin antibody.

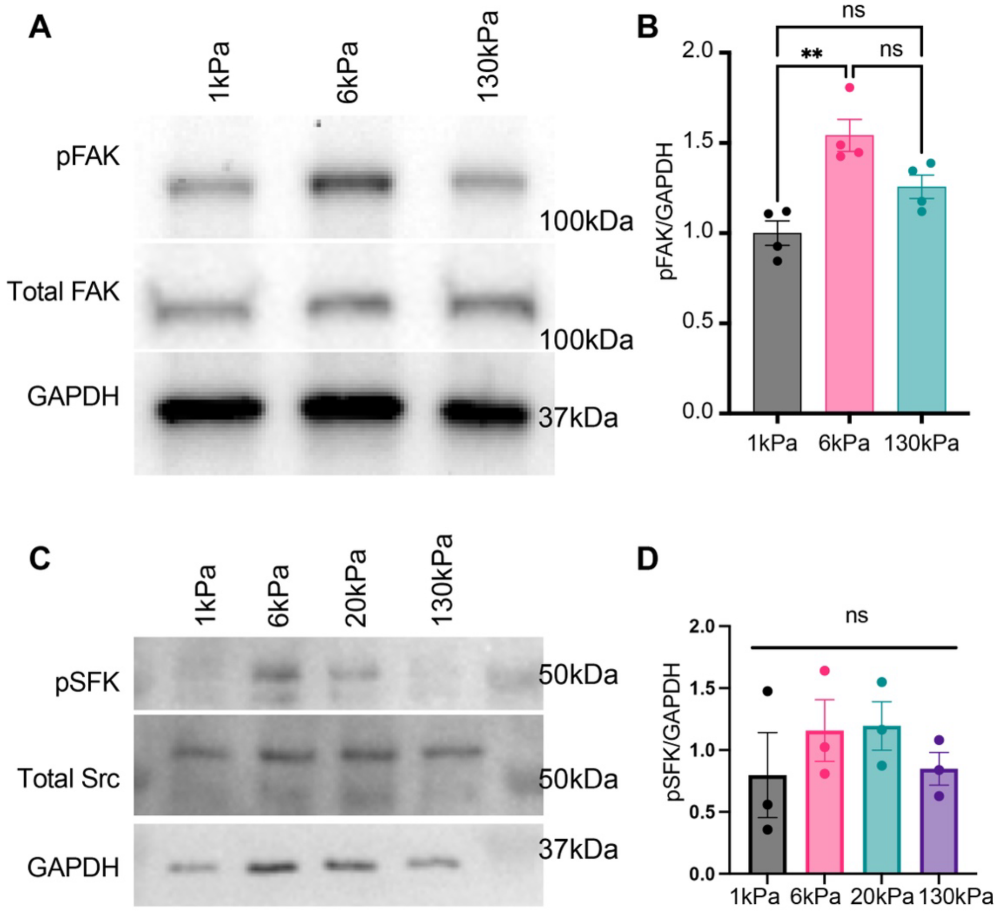

Supplementary Figure S5: FAK but not SFK activity is significantly increased at 6kPa. **A)** Neonatal rat cardiomyocytes were cultured on PDMS with the indicated stiffnesses, before lysis and blotting with pY397 FAK, total FAK and GAPDH antibodies. Quantified in **(B)** from four independent biological repeats. **C)** pSFK shows shows no significant stiffness-dependent difference. Noteworthy, the pSFK band does not overlap with Src. Quantified in **(D)** from three independent biological repeats. *p<0.05, **p<0.01, ***p<0.001, ****p<0.0001; p-values from one-way ANOVA with Tukey correction for multiple comparisons.

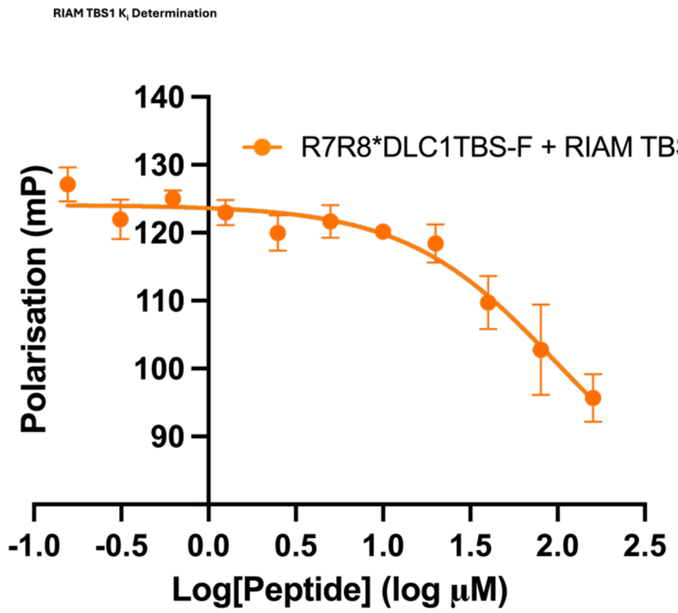

Supplemental Figure S6: RIAM TBS1 K_i determination from competition against DLC1 TBS-F bound to R7R8. Plot showing polarisation vs log[Peptide]. Data were fit using the GraphPad Prism Fit K_i equation with [DLC1 TBS-F] = 0.5 µM and $K_{d,DLC1TBS}$ = 4.92 µM, which produced $K_{i,RIAMTBS1}$ = 90 ± 48 µM.

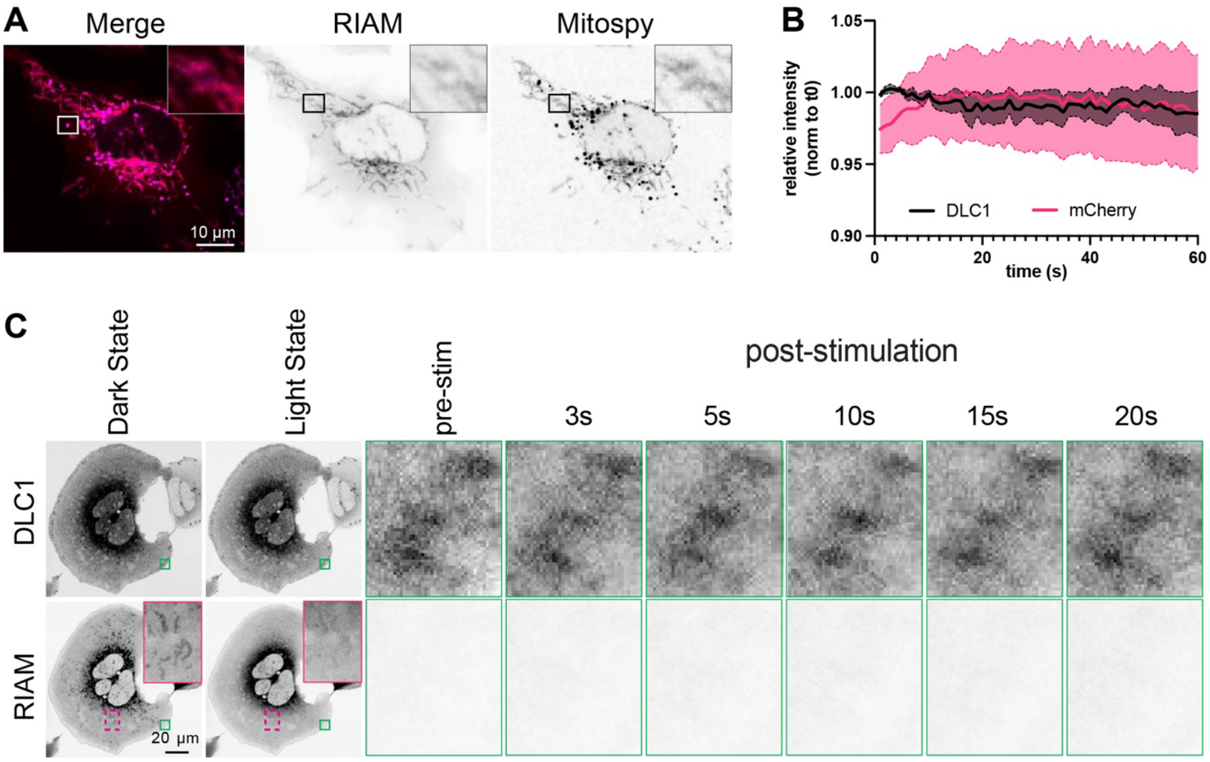

Supplementary Figure S7: LOVTRAP control experiments confirm localisation to mitochondria and specific competition for adhesion binding. A) C2C12 cells were transfected with mCherry-RIAM-ZDK and LOV-TOM20 and labelled with MitoSpy NIR-DilC1 to confirm mitochondrial targeting. **B,C)** C2C12 cells were transfected with mCherry-ZDK, DLC1-GFP and LOV-TOM20 and subjected to the same stimulation protocol as for the competition experiment in Figure 5.

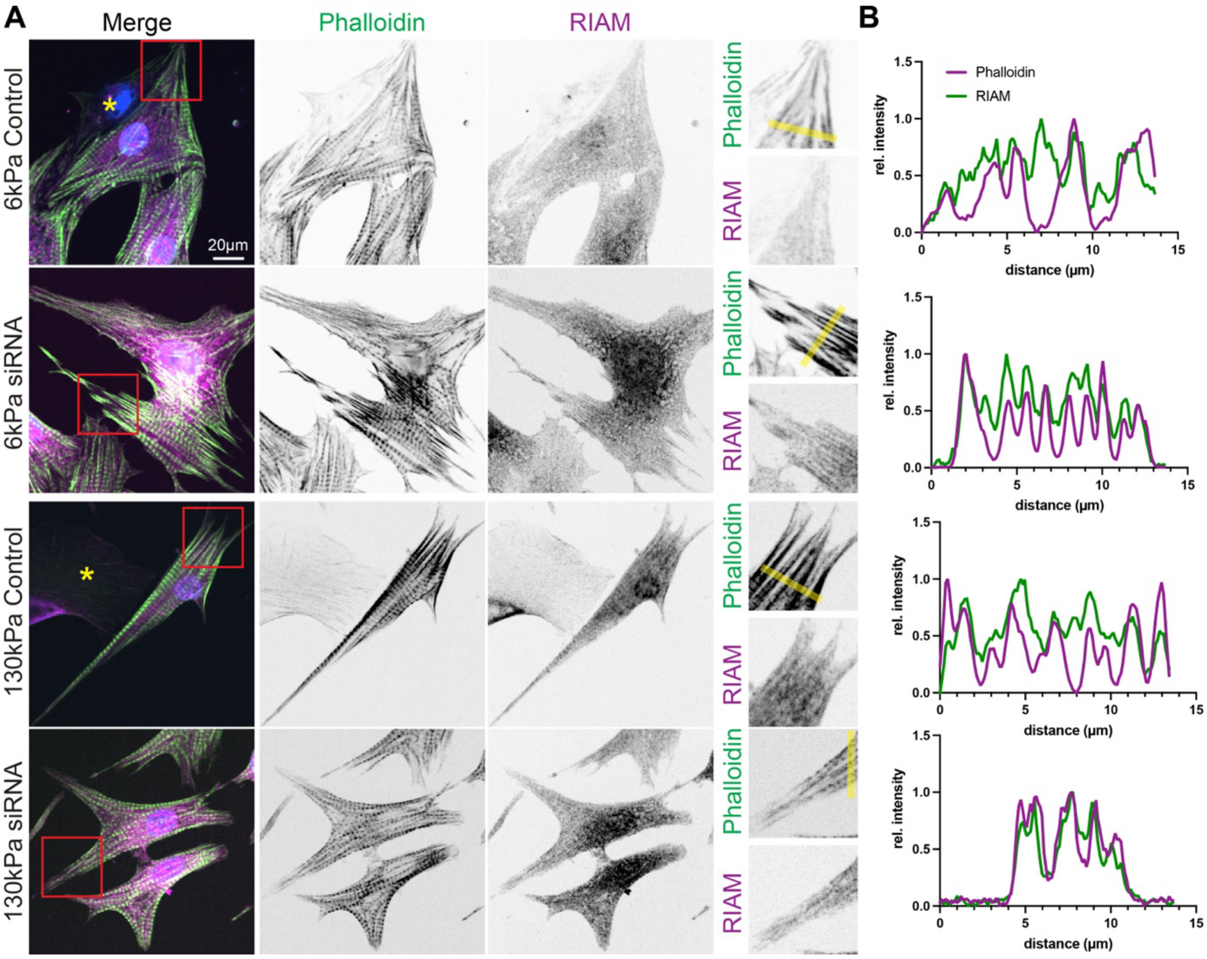

Supplementary Figure S8:RIAM localisation to cardiomyocyte adhesions is enhanced on 6kPa after DLC1 knockdown. A) Neonatal rat cardiomyocytes were treated with control or DLC1 siRNA and stained with an anti-RIAM antibody and Phalloidin. **B)** profile plots show relative intensities of RIAM and Phalloidin from the line indicated in yellow in panel A.

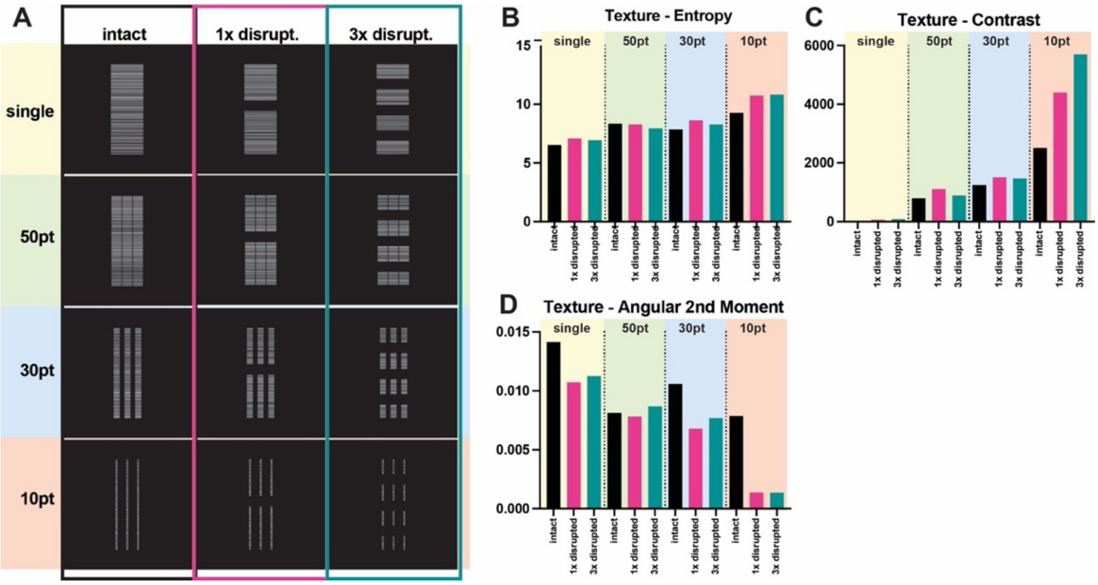

Supplementary Figure S9: Texture measurements for evaluating cardiomyocyte myofibril structure. Idealised cells were designed in the same scale as cell images from Figures 6 and Supplementary Figure S9. Cells were designed as shown in **(A)** including a different myofibrillar width and different extent of disruptions. Cells were analysed with the

same image analysis pipeline as used for microscopy images. The texture measurements for **(B)** 'Entropy', **(C)** 'Contrast', and **(D)** 'Angular 2nd Moment' showed good correlation with increasing loss of sarcomeric structure.

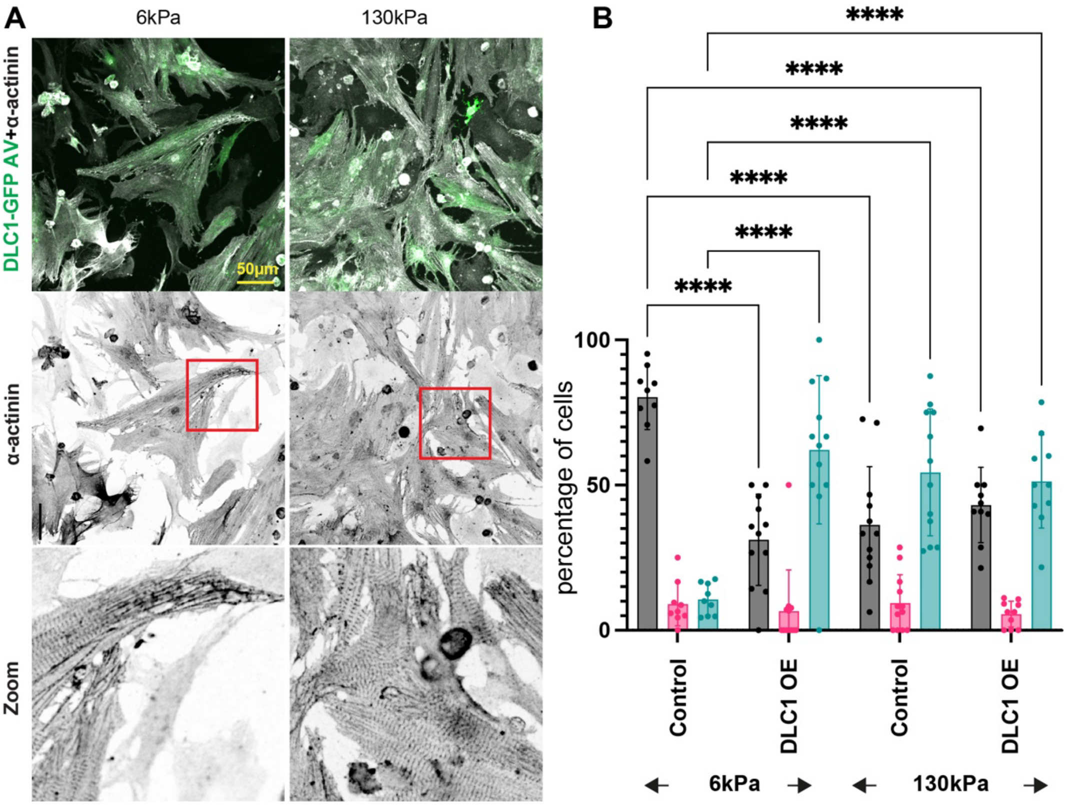

Supplementary Figure S10: DLC1 overexpression leads to increased stress-fibre like structures. A) Neonatal rat cardiomyocytes were cultured on PDMS with the indicated stiffness. Transduction with a DLC1-GFP adenoviral vector (DLC1 OE) results in increased stress-fibre like structures. Quantified in **(B)** from three independent biological repeats. *p<0.05, **p<0.01, ***p<0.001, ****p<0.0001; p-values from one-way ANOVA with Tukey correction for multiple comparisons.

Supplementary Tables:

Supplementary Table 1:

Clinical data of patients providing cardiac tissue for Western blots. HTx – heart transplant, AoS – aortic stenosis, LVAD – left ventricular assist device, DM – diabetes mellitus, NIDDM – non-insulin-dependent diabetes, IDDM – insulin dependent diabetes mellitus, COPD – chronic obstructive pulmonary disease, CAD – coronary artery disease, MI – myocardial infarction, EF – ejection fraction, SR – sinus rhythm, AF – atrial fibrillation , ICD – implantable cardioverter defibrillator, ECMO – extracorporeall membrane oxygenation, PMK/ICD – pacemaker/ implantable cardioverter, PTCA – percutaneous transluminal coronary angioplasty, Cx – circumflex artery, CABG – coronary artery bypass graft, AVR - aortic Valve Replacement

No.	Type	Sample region	Age at intervention	Hypertension	DM (0 = no, 1 = NIDDM, 2 = IDDM)	Dyslipidemia (0 = no, 1 = yes)	Smoke (0 = no, 1 = active smoker, 2 = former)	COPD	CAD	Previous MI	EF	Heart rhythm	Previous Cardiac Surgery	Valvular Disease	Cardiomyopathy	Operation
**2**	**HTx**	LV	38	0	0	0	0	0	0	0	27	SR	ICD implantation, ECMO	Severe Mitral Regurgitation	DCM	Heart Transplant
**3**	**HTx**	LV	63	0	0	0	0	0	0	0	30	AF	ICD implantation, Mitral Valve Replacement	0	DCM	Heart Transplant
**4**	**HTx**	LV	52	0	0	0	0	0	0	0	15	SR	ICD implantation, ECMO	0	ACM	Heart Transplant
**5**	**HTx**	LV	66	1	0	0	0	0	0	0	15	SR	ICD implantation	Moderate Mitral Regurgitation - Moderate Tricuspid Regurgitation	DCM	Heart Transplant
**6**	**HTx**	LV	64	1	0	0	2	0	0	0	20	SR	ICD implantation	Severe Mitral Regurgitation	DCM	Heart Transplant
**8**	**HTx**	LV	68	0	0	0	2	0	0	0	15	AF	ICD implantation	0	DCM	Heart Transplant
**9**	**LVAD**	LV apex	54	1	0	1	2	1	1	1	20	SR	ICD implantation	0	IHD	LVAD
**10**	**LVAD**	LV apex	57	1	0	0	0	0	1	1	18	SR	ICD implantation	0	IHD	LVAD
**11**	**LVAD**	LV apex	64	1	0	0	0	0	1	1	19	SR	ICD implantation	0	IHD	LVAD
**12**	**LVAD**	LV apex	59	0	0	0	0	0	0	0	19	SR	ICD implantation	0	DCM	LVAD
**13**	**LVAD**	LV apex	48	1	1	1	0	0	1	1	20	SR	ICD implantation	0	IHD	LVAD
**17**	**LVAD**	LV apex	66	1	1	1	0	0	0	0	36	Paroxismal AF	ICD implantation	0	DCM	LVAD
**18**	**AoS**	LV	71	1	0	1	2	0	1	1	27	SR	PMK/ICD implantation	Severe Aortic Stenosis	0	AVR + 1 CABG
**23**	**AoS**	LV	64	1	0	1	0	0	1	0	55	SR	PTCA on Cx	Severe Aortic Stenosis	0	AVR
**24**	**AoS**	LV	56	1	0	1	0	0	0	0	71	SR	0	Severe Aortic Stenosis	0	AVR + subaortic membrane removal
**28**	**AoS**	LV	72	1	0	1	0	0	1	0	57	SR	0	Severe Aortic Stenosis	0	AVR + 1 CABG
**32**	**AoS**	LV	78	1	0	1	0	0	1	0	55	SR	0	Severe Aortic Stenosis	0	AVR + 2 CABG
**33**	**AoS**	LV	82	1	0	0	0	0	1	0	65	SR	0	Severe Aortic Stenosis	0	AVR + 2 CABG
